# Leveraging the COVID-19 pandemic as a natural experiment to assess changes in antibiotic use and antibiotic-resistant *E. coli* carriage in semi-rural Ecuador

**DOI:** 10.1038/s41598-023-39532-5

**Published:** 2023-09-08

**Authors:** Heather K. Amato, Fernanda Loayza, Liseth Salinas, Diana Paredes, Daniela García, Soledad Sarzosa, Carlos Saraiva-Garcia, Timothy J. Johnson, Amy J. Pickering, Lee W. Riley, Gabriel Trueba, Jay P. Graham

**Affiliations:** 1 Environmental Health Sciences Division, School of Public Health, University of California, Berkeley, CA USA; 2 Present Address: Department of Civil and Environmental Engineering, University of California, Berkeley, CA USA; 3 Instituto de Microbiología, Colegio de Ciencias Biológicas y Ambientales, Universidad San Francisco de Quito, Quito, Pichincha Ecuador; 4 Department of Veterinary and Biomedical Sciences, University of Minnesota, Saint Paul, MN USA; 5 Blum Center for Developing Economies, University of California, Berkeley, CA USA; 6 Chan Zuckerberg Biohub, San Francisco, CA USA; 7 Division of Infectious Diseases and Vaccinology, School of Public Health, University of California, Berkeley, CA 94720 USA

**Keywords:** Antimicrobials, Applied microbiology, Bacteria, Pathogens, Policy and public health in microbiology, Infectious diseases, Microbiology, Diseases, Health care, Patient education, Public health

## Abstract

The coronavirus 2019 (COVID-19) pandemic has had significant impacts on health systems, population dynamics, public health awareness, and antibiotic stewardship, which could affect antibiotic resistant bacteria (ARB) emergence and transmission. In this study, we aimed to compare knowledge, attitudes, and practices (KAP) of antibiotic use and ARB carriage in Ecuadorian communities before versus after the COVID-19 pandemic began. We leveraged data collected for a repeated measures observational study of third-generation cephalosporin-resistant *E. coli* (3GCR-EC) carriage among children in semi-rural communities in Quito, Ecuador between July 2018 and September 2021. We included 241 households that participated in surveys and child stool sample collection in 2019, before the pandemic, and in 2021, after the pandemic began. We estimated adjusted Prevalence Ratios (aPR) and 95% Confidence Intervals (CI) using logistic and Poisson regression models. Child antibiotic use in the last 3 months declined from 17% pre-pandemic to 5% in 2021 (aPR: 0.30; 95% CI 0.15, 0.61) and 3GCR-EC carriage among children declined from 40 to 23% (aPR: 0.48; 95% CI 0.32, 0.73). Multi-drug resistance declined from 86 to 70% (aPR: 0.32; 95% CI 0.13; 0.79), the average number of antibiotic resistance genes (ARGs) per 3GCR-EC isolate declined from 9.9 to 7.8 (aPR of 0.79; 95% CI 0.65, 0.96), and the diversity of ARGs was lower in 2021. In the context of Ecuador, where COVID-19 prevention and control measures were strictly enforced after its major cities experienced some of the world’s the highest mortality rates from SARS-CoV-2 infections, antibiotic use and ARB carriage declined in semi-rural communities of Quito from 2019 to 2021.

## Introduction

Among the wide-ranging public health issues significantly impacted by the Coronavirus Disease 2019 (COVID-19) pandemic, one of the most urgent is the global crisis of antibiotic resistance. Healthcare systems, antibiotic usage, and infection control and prevention measures have changed significantly due to the pandemic, potentially resulting in short-term and long-term impacts on antibiotic resistance^1^. Changes in the dynamics of antibiotic resistant bacteria (ARB) emergence and transmission are still unfolding, and findings to-date suggest complex interactions between cross-sectoral impacts of COVID-19 and antibiotic resistance.

The COVID-19 pandemic strained healthcare systems across the globe, most severely impacting low- and middle-income countries (LMICs) where health systems were already under-resourced. Healthcare systems in LMICs often have a limited capacity to serve large populations, an insufficient healthcare workforce, limited diagnostic testing capacity, and inadequate public health insurance—all of which were exacerbated by the pandemic^2–4^. These cross-cutting challenges can lead to excessive and inappropriate antibiotic prescribing among healthcare providers, a strong reliance on unregulated, over-the-counter (OTC) access to antibiotics, and suboptimal adherence^5^.

Additional challenges include limited antibiotic stewardship and the convenience of self-medication in LMICs^6–8^. Prior to the pandemic, antibiotics were prescribed—largely unnecessarily—to treat an estimated 80% of respiratory infections and 50% of diarrheal infections in children in LMICs^9^. Individuals commonly sought antibiotics from pharmacies, friends, and family members early in the pandemic due to uncertainty and anxiety about COVID-19, and pharmacists regularly sold antibiotics OTC^10–13^. Access to the right antibiotics when appropriate is critical for preventing severe illness, but inappropriate use can increase the length of infection, disease severity, and risk of developing a drug-resistant infection and further complications^14^. While antibiotic use in high-income countries like the United States decreased overall throughout the pandemic^15^, data on antibiotic use and resistance in LMICs before versus after the start of the pandemic is urgently needed.

Excessive and inappropriate antibiotic use among COVID-19 patients is well-documented. Broad-spectrum and last-line antibiotics were prescribed to more than half of patients with SARS-CoV-2 infections in several high-income countries, as well as China, India, and Brazil, despite low rates of bacterial co-infections (< 10%)^16, 17^. Among patients with co-infections or secondary bacterial infections, multidrug-resistant (MDR) infections are common, including extended- spectrum beta-lactamase producing Enterobacterales (ESBL-E)^18–21^. ESBL-E are resistant to cephalosporins—a group of beta-lactam antibiotics commonly used to treat life-threatening infections—and are often MDR, resulting in limited treatment options and high mortality rates^22–25^. While excessive antibiotic use and ESBL-E infections among COVD-19 patients is concerning, it is unclear whether antibiotic use and ESBL-E carriage also increased in community settings during the pandemic.

Community-acquired ESBL-E and MDR infection prevalence is increasing globally and is of particular concern in LMICs, where inappropriate use of antibiotics, inadequate water and sanitation infrastructure, and poor community hygiene contribute to the spread of ARB in the environment, in animals, and in humans^26–29^. While inappropriate and unregulated antibiotic use may increase the risk of ESBL-E and MDR emergence, bans on international travel, restricted local movement, school and business closures, and improved community hygiene and infection control as a result of the COVID-19 pandemic may reduce the risk of community-acquired ARB transmission^1, 30^. Public health recommendations and mandates—like frequent hand washing and mask-wearing—also have the potential to prevent community-acquired ARB infections^31, 32^. The direction and magnitude of changes in community-acquired antibiotic resistance in the context of a global pandemic, with complex factors influencing infectious disease dynamics, can be estimated by leveraging the COVID-19 pandemic as a natural experiment.

Ecuador experienced some of the deadliest COVID-19 outbreaks in the world, with an estimated 36,922 excess deaths between March 17 and October 22, 2020^33^. Early in the pandemic, Ecuadorian hospitals in major cities (Guayaquil and Quito) were overwhelmed with COVID-19 patients. Several initial steps were taken to reduce COVID-19 transmission and bolster the strained healthcare system. A call center was established in late February 2020 to begin tracking reports of COVID-19 symptoms, inform symptomatic people of isolation guidelines, and provide in-person care. A national State of Sanitary Emergency was issued on March 11, 2020, after the World Health Organization (WHO) declared COVID-19 a global pandemic. The government closed the national borders, restricted local travel by license plate number, closed schools, workplaces, and public transit, banned large gatherings, and issued a strict curfew from 2pm to 5am^34^. Communications campaigns were launched to encourage hand washing, use of face masks, and social distancing for the prevention and control of COVID-19^34^. Wearing face masks in public spaces became mandatory as of April 2020, a ruling that remained in effect until April 2022^35^. Strict lockdowns remained in place in Quito and Guayaquil until May 2020, when some restrictions on businesses and public and private transit were lifted^36^.

National and local curfews have been implemented in Ecuador at various times since the beginning of the pandemic based on trends in cases and hospitalizations. A December 21, 2020-January 4, 2021 curfew prohibited everyone from leaving their home during curfew hours (10pm–4am; excluding essential workers), prohibited all gatherings of more than 10 people, and included beach closures on holidays. Schools were also closed and transit between provinces was allowed at 75% capacity. Business hours were restricted to 8am–2pm with a maximum capacity of 50% (30% for hotels and restaurants; bars were forced to close). To further restrict unnecessary travel and reduce the risk of exposure, vehicles were only allowed on the road every other day based on their license plate number. Land and sea borders were also closed during this time. International air travelers from certain regions with high COVID-19 transmission were required to present negative polymerase chain reaction (PCR) COVID-19 test results in addition to a negative antigen test at the airport, while all international travelers were required to quarantine after arriving in Ecuador^37^.

To characterize how the COVID-19 pandemic impacted community-acquired ARB transmission in an upper-middle-income country, we leveraged data from a longitudinal observational study, which took place before and after the onset of the pandemic, on ESBL-E carriage among healthy children in Quito, Ecuador. The objective of this study was to compare caregiver knowledge, attitudes, and practices (KAP) around antibiotic use and antibiotic-resistant *E. coli* carriage in healthy children before versus after the onset of the COVID-19 pandemic.

## Methods

We analyzed a subset of data collected for a repeated measures observational study conducted in semi-rural communities east of Quito, Ecuador between July 2018 and September 2021. The parent study enrolled 605 households with at least one child between 6 months and 5 years old at the time of enrollment throughout the study period. A single child was enrolled per household. Child fecal samples and survey data on child and household characteristics, including caregiver-reported child illness, antibiotic use, and KAP around antibiotics, were collected at up to five time points per household throughout the study period (Supplemental Materials, Fig. S1). After the third data collection cycle in July through November of 2019, the fourth of five data collection cycles began in January 2020 and ended prematurely in March 2020 due to initial lockdowns at the beginning of the COVID-19 pandemic. Data collection resumed for the fifth cycle in April 2021 through September 2021.

Details of the parent study design and methodology are described elsewhere (Amato et al. 2023^38^, Accepted, PLOS Medicine).

### Patient and public involvement

Antibiotic resistance is an issue that our communities were unfamiliar with prior to beginning the study, especially because it can spread asymptomatically. Thus, we worked with a community organizer to identify community needs we could fulfill while simultaneously achieving research priorities of microbiology and public health researchers at the Universidad San Francisco de Quito and University of California, Berkeley. For example, we provided individual results on child intestinal parasites to caregivers, which were of interest to the community. We also modified the study incentive based on community feedback; we planned to distribute hygiene kits but instead provided basic items like rice and beans, as requested by community members.

For the design of the study, we involved a community organizer who lives in the community and meets regularly with community residents. Based on feedback from residents communicated to us via the community organizer, we dropped some survey questions that were contextually irrelevant or inappropriate. We also improved our process for delivering study results back to households by working with a community organizer; we reduced turnaround time for communicating child intestinal parasite data to caregivers, along with recommendations for seeking treatment. Community residents were also included as part of the study team. These individuals have long standing relationships with the community and have worked on several public health campaigns by the government and by local NGOs. We relied heavily on these members of our research team who live in the community to guide us and change protocols and adjust to timelines that were acceptable to the community.

We have hosted two community-wide events (all study participants invited) to share and discuss results with the community. We are also working to design health promotion materials that are collaboratively designed with feedback from the community. The community events were a great success and helped build a stronger understanding of zoonotic infectious diseases and antibiotic resistance (see short video of one event here: https://vimeo.com/654315270).

### Study site

The study area is approximately 320 km^2^ spanning seven semi-rural parishes east of Quito, Ecuador. Three parishes have high-intensity commercial food-animal production (> 30 operations) and approximately 30% of households in the parent study owned backyard food-animals, known risk factors for antibiotic-resistant infections (Amato et al. In Review). Household-level access to water, sanitation, and hygiene infrastructure is very high in this study area. In the parent study, almost all households had handwashing stations with both soap and water, flush toilets, and piped drinking water inside the home. There are no wastewater treatment plants in the study area; wastewater is discharged directly into surface water.

### Microbiological methods

Child fecal samples were plated on MacConkey agar (Difco, Sparks, Maryland) with 2 mg/L of ceftriaxone and incubated for 18 h at 37 °C to screen for 3GCR-EC (Salinas et al., 2019). One to five isolates phenotypically matching *E. coli* were selected from each fecal sample and preserved at  − 80 °C in Trypticase Soy Broth medium (Difco, Sparks, MD) with 20% glycerol. Isolates were thawed and regrown on MacConkey agar at 37 °C for 24 h for evaluation of antibiotic susceptibility by the disk diffusion method (Kirby Bauer test) on Mueller–Hinton agar (Difco, Sparks, Maryland). Colonies were inoculated onto Chromocult® coliform agar to confirm presumptive *E. coli* growth (Merck, Darmstadt, Germany).

### Antibiotic susceptibility testing

Antibiotic susceptibility testing of all 3GCR-EC isolates was conducted for 10 antibiotics: ampicillin (AM; 10 μg), ceftazidime (CAZ; 30 μg), ciprofloxacin (CIP; 5 μg), cefotaxime (CTX; 30 μg), cefazolin (CZ; 30 μg), cefepime (FEP; 30 μg), gentamicin (GM; 10 μg), imipenem (IPM; 10 μg), trimethoprim/sulfamethoxazole (SXT; 1.25 per 23.75 μg), and tetracycline (TE; 30 μg). Isolates were identified as either susceptible or resistant to each antibiotic according to the resistance or susceptibility interpretation criteria from Clinical and Laboratory Standards Institute (CLSI) guidelines^39^. *E. coli* ATCC 25922 was used as the quality control strain. Multidrug resistance was determined based on the number of macro-classes to which each isolate was resistant. Macro-classes were defined as cephalosporin/beta-lactamase inhibitors, penicillins, aminoglycosides, carbapenems, fluoroquinolones, tetracyclines, and folate pathway inhibitors. 3GCR-EC isolates from the same fecal sample with identical phenotypic resistance profiles were considered duplicates and were deduplicated prior to analyses. If multiple 3GCR-EC isolates per fecal sample remained after deduplication, we selected the first isolate per sample to be included in this analysis.

### DNA sequencing and analysis

We extracted purified DNA from the isolates using Wizard® Genomic DNA Purification (Promega) kits and QIAGEN© DNEasy Blood & Tissue Kits according to the manufacturer’s instructions. Whole-genome sequencing of *E. coli* isolates was carried out at the University of Minnesota using Illumina MiSeq with Nextera XT libraries. Raw reads were quality-trimmed and adapter-trimmed using trimmomatic^40^ and assembled using SPAdes (Bankevich et al. 2012). Antibiotic resistance genes (ARGs) were detected using ABRicate (version 0.813) and a curated version of the ResFinder database (Zankari et al. 2012). We also performed in silico multilocus sequence typing (MLST) based on seven housekeeping genes (*adk*, *fumC*, *gyrB*, *icd*, *mdh*, *purA*, and *recA*), an additional eight housekeeping genes (*dinB*, *icdA*, *pabB*, *polB*, *putP*, *trpA*, *trpB*, and *uidA*), and core genome (cgMLST) using MLST 2.0^41^ and cgMLSTFinder 1.1^42^. Detailed methods are previously described^43^.

### Statistical analyses

For this before and after analysis, we compared caregiver KAP, child health and antibiotic use, and 3GCR-EC carriage in children from data collected in the third cycle in 2019 (before COVID-19 began) to data collected in the fifth cycle in 2021 (after COVID-19 began) (Fig. S1). We used a within subjects design, including only data from households that participated in data collection in both 2019 and 2021, so each household served as its own control in the comparison. Households that did not participate in both the 2019 and 2021 data collection cycles were excluded from this analysis. We estimated the change in caregiver KAP around antibiotic use; caregiver reported child antibiotic use and child illness; child 3GCR-EC carriage; and phenotypic and genotypic resistance to antibiotics associated with the onset of the COVID-19 pandemic. Multivariable logistic and Poisson regression models were used to calculate adjusted prevalence ratios (aPRs) for each outcome, with the time of the observation (pre-pandemic in 2019 vs. 2021, after COVID-19 began) as the independent variable. We included pre-specified covariates (child age, child sex, caregiver education, and household wealth) to control for confounders and strong predictors of the outcomes^44^. Household wealth was measured using a principal component score estimated from asset ownership variables. Robust standard errors were estimated with generalized estimating equations and an exchangeable working correlation to adjust for repeated measures at the household/child level. Statistical analyses and visualizations were completed in R version 3.6.1^45^ using the *dplyr*^46^, *arsenal*^47^, *geepack*^48^, and *ggplot2*^49^ packages.

### Sensitivity analyses

To assess whether the observed changes in our outcomes of interest were possibly due to other secular trends, we conducted sensitivity analyses using the differences-in-differences approach, a quasi-experimental, controlled before-and-after design^50^. We compared the changes from data collection cycle one in 2018 to cycle three in 2019 (comparison 1: secular trends pre-COVID-19) with the changes from cycle three in 2019 to cycle five in 2021 (comparison 2: pre versus post pandemic onset) (Fig. S1). We used multivariable logistic and Poisson regression models with pre-specified covariates (child age, child sex, caregiver education, household wealth, and parish), plus an interaction term for the time and comparison (i.e., treatment) group. For this sensitivity analysis, we included all child fecal samples and multiple isolates per fecal sample when applicable, regardless of their participation in each data collection cycle.

### Ethics approval and consent to participate

The study was approved by the Office for Protection of Human Subjects (OPHS) at the University of California, Berkeley (IRB# 2019-02-11803) and by the Bioethics Committee at the Universidad San Francisco de Quito (#2017-178 M), and the Ecuadorian Health Ministry (#MSPCURI000243-3). All study participants provided informed consent prior to participation. Caregivers provided written assent prior to their child’s participation in the study.

## Results

For the main analysis, we included 241 children from households that participated in both the third (pre-pandemic, 2019) and fifth (post-pandemic onset, 2021) data collection cycles. Forty-two percent (42%) of children were female. In 2019, 71% of children were at least two years of age and the mean age was 3.4 (Standard Deviation (SD): 1.6). The mean age of caregivers who responded to household surveys was 32.8 (SD: 12.0), and 73% of caregivers had at least a high school or college education. Of note, 74% of children had contact with pets in the last three months in both 2019 and 2021 (Table S3). Twelve percent (12%) of children attended daycare prior to the pandemic, while only 4% attended daycare in 2021 after the pandemic began (Table S3).

### Caregiver knowledge, attitudes, and practices

**Figure 1:**
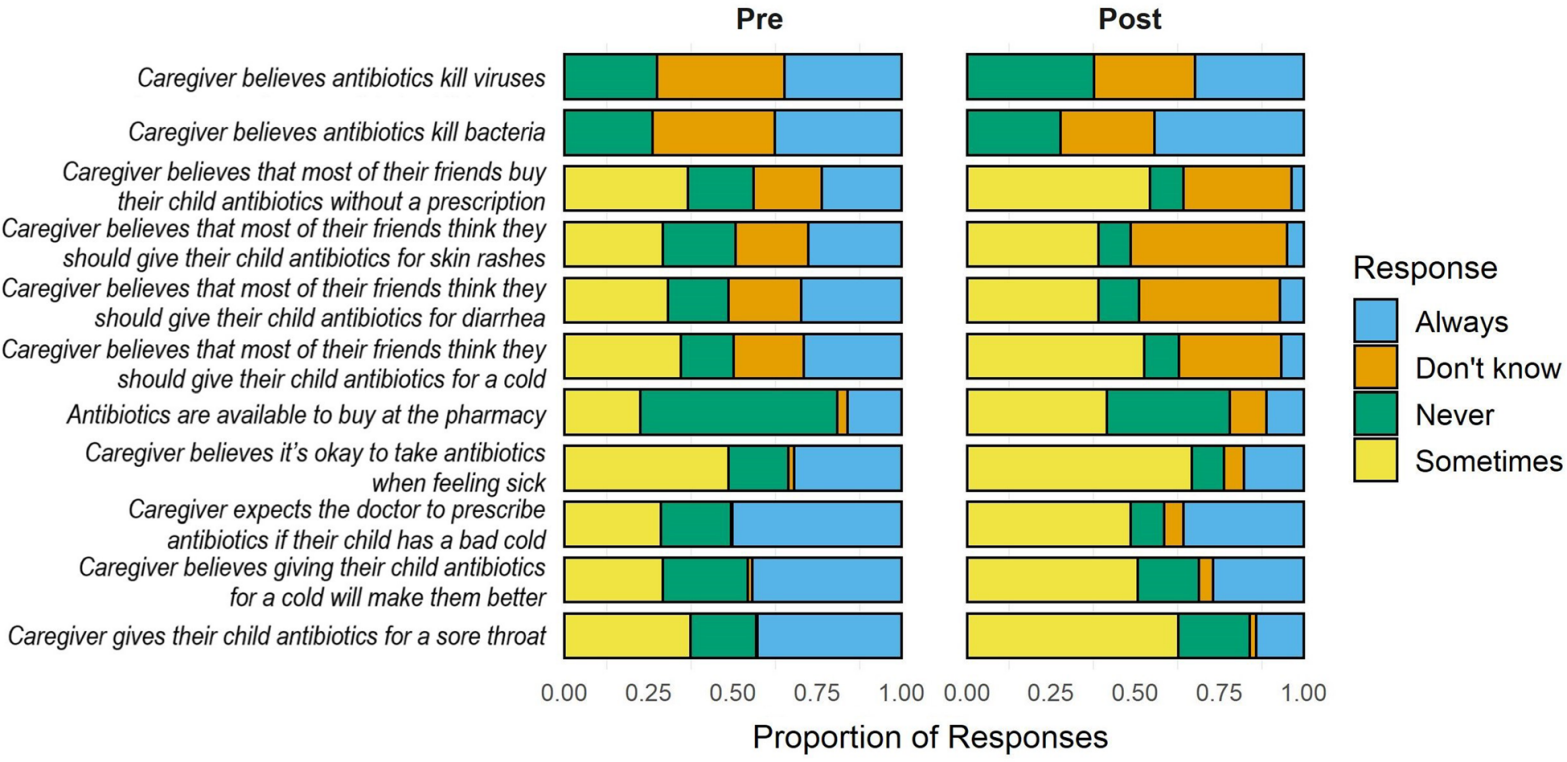
Caregiver knowledge, attitudes, and practices around antibiotic use before the pandemic in 2019 ("Pre") and in 2021 after the COVID-19 pandemic began ("Post").

Caregiver knowledge that antibiotics do not kill viruses increased from 27% in 2019 to 38% in 2021 (Fig. 1, Table S1). Fewer caregivers responded “yes” or “don’t know” in 2021 when asked if antibiotics kill viruses (adjusted Prevalence Ratio (aPR): 0.54; 95% Confidence Interval (CI) 0.35, 0.86) (Table 1). In 2021, fewer caregivers responded “don’t know” and more caregivers correctly responded “yes” when asked if antibiotics kill bacteria compared to 2019, before the pandemic (Fig. 1). However, the change in the proportion of incorrect responses (“no” or “don’t know”) did not change significantly (aPR: 0.73; 95% CI 0.48, 1.10) (Table 1). In sensitivity analyses of a total of 590 households, the changes in knowledge about antibiotic use observed before COVID-19 (from 2018 to 2019) were not significantly different from the changes observed after vs. before COVID-19 began (Table S6). However, the proportion of caregivers who correctly stated that antibiotics do not kill viruses rose from 25 to 28% in 2018 and 2019, then jumped to 36% in 2021 (Table S5).

**Table 1:** Prevalence ratios of caregiver knowledge, attitudes, and practices around antibiotic use in 2021 after the COVID-19 began compared to 2019 before the pandemic.

Category	Outcome variable	N	aPR*(95% CI)	*P-*value
Caregiver knowledge about antibiotics	Caregiver believes antibiotics kill bacteria (ref = yes)	480	0.73 (0.48, 1.10)	0.1316
	Caregiver believes antibiotics kill viruses (ref = no)	480	0.54 (0.35, 0.86)	0.0087
Caregiver attitudes about antibiotic use (ref = sometimes)	Caregiver gives their child antibiotics for a sore throat	480	0.37 (0.24, 0.57)	< 0.0001
	Caregiver believes giving their child antibiotics for a cold will make them better	480	0.42 (0.27, 0.65)	0.0001
	Caregiver expects the doctor to prescribe antibiotics if their child has a bad cold	480	0.45 (0.29, 0.70)	0.0004
	Caregiver believes it’s okay to take antibiotics when feeling sick	480	0.48 (0.33, 0.71)	0.0002
	Antibiotics are available to buy at the pharmacy	480	0.44 (0.28, 0.70)	0.0004
Caregiver perceptions of social norms around antibiotic use (ref = sometimes)	Caregiver believes that most of their friends think they should give their child antibiotics for a cold	479	0.51 (0.34, 0.77)	0.0012
	Caregiver believes that most of their friends think they should give their child antibiotics for diarrhea	480	0.74 (0.48, 1.13)	0.1654
	Caregiver believes that most of their friends think they should give their child antibiotics for skin rashes	480	0.66 (0.43, 1.01)	0.0582
	Caregiver believes that most of their friends buy their child antibiotics without a prescription	480	0.52 (0.35, 0.78)	0.0014

*Adjusted Prevalence Ratios (aPR) estimated with logistic regressions using Generalized Estimating Equations to adjust for repeated measures, and adjusted for confounding by including the following covariates: child age, child sex, household wealth (asset score), and caregiver education. 95% CI 95% Confidence Intervals.

Caregiver attitudes and practices around antibiotic use changed, becoming more nuanced after the COVID-19 pandemic began. The proportion of caregivers responding “don’t know” increased after COVID-19 across all questions about attitudes, practices, and social norms around antibiotic use (Fig. 1). When asked about situations in which the caregiver or their child should take antibiotics, whether they can expect to purchase antibiotics at the pharmacy or receive a prescription from their doctor, and whether taking antibiotics will make them feel better, caregivers responded “always” or “never” less frequently in 2021 compared to in 2019 (Fig. 1). For example, 44% of caregivers believed that giving their child antibiotics for a cold would “always” make them better before the pandemic; this dropped to 27% after the pandemic (aPR: 0.42; 95% CI 0.27, 0.65) (Table 1). For this same question, 29% believed antibiotics would “sometimes” make their child feel better before the pandemic, which increased to 51% after the pandemic (Fig. 1). Changes in attitudes and practices around antibiotic use were only observed in before and after COVID-19 comparisons, while they remained largely unchanged from 2018 to 2019 before COVID-19 began based on sensitivity analyses (Table S5, Table S6).

Similar patterns were observed for caregiver-perceived social norms around antibiotic use. Before COVID-19, 29% of caregivers believed that most of their friends think they should “always” give their child antibiotics for a cold, while 16% believed their friends think they should “never” give their child antibiotics for a cold (Fig. 1). These responses declined to 7% and 10%, respectively, after the pandemic (aPR: 0.51; 95% CI 0.34, 0.77), while the proportion of caregivers who responded “sometimes” increased from 34 to 53% (Table 1, Fig. 1). Notably, the proportion of caregivers who believed most of their friends “always” buy their child antibiotics without a prescription decreased from 24% before COVID-19 to 4% in 2021 (Fig. 1). In sensitivity analyses, the difference between the changes in perceptions of social norms around antibiotic use from 2019 to 2021 and changes observed from 2018 to 2019 was not statistically significant (Table S6). However, the ratios of aPRs remained < 1, indicating a greater decrease in “always” and “never” responses from 2019 to 2021 compared to pre-COVID-19 trends. For example, caregivers included in the sensitivity analysis said they believed most of their friends should “always” give their child antibiotics for a cold 24% of the time in 2018, 27% of the time in 2019, and only 6% of the time in 2021 (Table S5). This trend was observed in sensitivity analyses for all variables on caregiver perceived social norms around antibiotic use.

### Child health and antibiotic use

**Figure 2:**
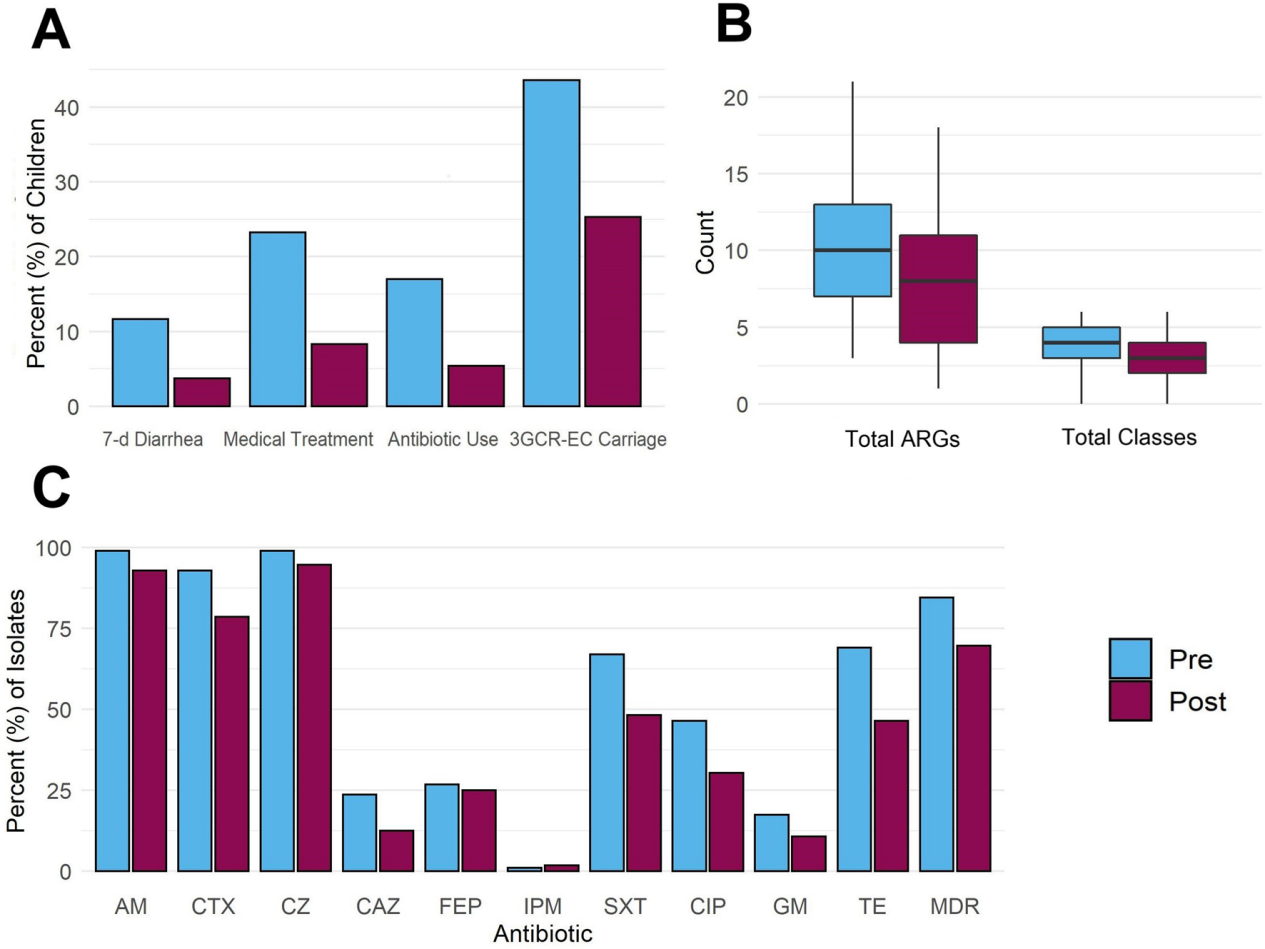
Percent of children (N = 241) with caregiver-reported 7-d diarrhea, medical treatment in the last 3 months, antibiotic use in the last 3 months, and current 3GCR-EC carriage (**A**); number of drug classes to which each 3GCR-EC isolate was phenotypically resistant (N = 153) and total number of antibiotic resistance genes (ARGs) per isolate among sequenced 3GCR-EC isolates (N = 135) (**B**); and percent of all 3GCR-EC isolates (N = 153) expressing phenotypic resistance to specific antibiotics (**C**) before and after the COVID-19 pandemic began. 3GCR-EC: third-generation cephalosporin-resistant *E. coli*; AM: ampicillin; CAZ: ceftazidime; CIP: ciprofloxacin; CTX: cefotaxime; CZ: cefazolin; FEP: cefepime; GM: gentamicin; IPM: imipenem; SXT: trimethoprim/sulfamethoxazole; TE: tetracycline; MDR: multidrug-resistant (3 + classes).

Fewer children had diarrhea, received medical treatment, or took antibiotics after the onset of COVID-19 compared to before (Fig. 2). The proportion of children with caregiver-reported 7-d diarrhea decreased from 12% in 2019 to 4% in 2021 (aPR: 0.37; 95% CI 0.17, 0.81) (Table 2, Table S2). Enteric colonization of 3GCR-EC among children was also lower in 2021 compared to 2019 (Fig. 2). Forty percent (40%) of children were carriers of 3GCR-EC before COVID-19 and 23% carried 3GCR-EC after COVID-19 began (aPR: 0.48; 95% CI 0.32, 0.73) (Table 2, Table S2). The prevalence of child diarrhea, antibiotic use, and carriage of 3GCR-EC were already decreasing pre-COVID-19 (Table S5). However, reductions in the proportion of children who received medical treatment, took antibiotics, or were colonized with 3GCR-EC were significantly greater when comparing changes from 2019 to 2021 vs. pre-pandemic changes from 2018 to 2019 (Table S6).

**Table 2:** Prevalence ratios of 3GCR-EC carriage and phenotypic and genotypic antibiotic resistance among 3GCR-EC isolates from child fecal samples collected in 2021 after the COVID-19 pandemic began compared to samples collected in 2019 before the pandemic.

Category	Outcome variable	N	aPR* (95% CI)	*P-*value
Caregiver-reported child illness and antibiotic use (ref = no)	Child had diarrhea in the last 7 days	478	0.37 (0.17, 0.81)	0.0137
	Child received medical treatment in the last 3 months	480	0.30 (0.17, 0.53)	< 0.0001
	Child took an antibiotic in the last 3 months	479	0.30 (0.15, 0.61)	0.0009
Carriage of 3GCR-EC (ref = no)	3GCR-EC carriage	476	0.49 (0.33, 0.74)	0.0007
Phenotypic resistance among 3GCR-EC isolates (ref = susceptible)	Ampicillin (AM)	153	0.19 (0.02, 1.78)	0.1450
	Cefotaxime (CTX)	153	0.35 (0.12, 1.02)	0.0535
	Cefazolin (CZ)	153	0.26 (0.02, 2.80)	0.2683
	Ceftazidime (CAZ)	153	0.44 (0.16, 1.25)	0.1237
	Cefepime (FEP)	153	0.79 (0.35, 1.79)	0.5753
	Imipenem (IPM)	153	–	–
	Trimethoprim/sulfamethoxazole (SXT)	153	0.48 (0.23, 1.00)	0.0493
	Ciprofloxacin (CIP)	153	0.59 (0.28, 1.26)	0.1744
	Gentamicin (GM)	153	0.54 (0.19, 1.57)	0.2590
	Tetracycline (TE)	153	0.36 (0.16, 0.77)	0.0088
	MDR (3 + classes)	153	0.32 (0.13, 0.79)	0.0088
	Total classes	153	0.83 (0.73, 0.95)	0.0044
Abundance of ARGs (ref = no change)	Total ARGs	135	0.79 (0.65, 0.96)	0.0202

*AM* Ampicillin; *CAZ* Ceftazidime; *CIP* Ciprofloxacin; *CTX* Cefotaxime; *CZ* Cefazolin; *FEP* Cefepime; *GM* Gentamicin; *IPM* Imipenem; *SXT* Trimethoprim/sulfamethoxazole; *TE* Tetracycline. *MDR* Multidrug resistant (3 + classes).

*Adjusted Prevalence Ratios (aPR) estimated with logistic regressions (Poisson for Total Classes and Total ARGs) using Generalized Estimating Equations to adjust for repeated measures, and adjusted for confounding by including the following covariates: child age, child sex, household wealth (asset score), and caregiver education. (– indicates model did not converge due to small numbers.) 95% CI 95% Confidence Intervals.

### Antibiotic resistance of 3GCR-EC

Among 153 3GCR-EC colonies isolated before and after the beginning of the COVID-19 pandemic, almost all were resistant to the third-generation penicillin AM (97%) and first-generation cephalosporin CZ (97%) (Fig. 2, Table S2). Resistance to CTX and CAZ, both third-generation cephalosporins, declined from 93 to 79% (aPR: 0.35; 95% CI 0.12, 1.02) and from 24 to 13% (aPR: 0.44; 95% CI 0.16, 1.25), respectively (Fig. 2, Table 2). In before and after COVID-19 comparisons, there was no change in the proportion of isolates resistant to fourth-generation cephalosporin FEP, which was 26% overall (Fig. 2). However, the prevalence of FEP resistance in sensitivity analyses declined from 44% (n = 121/273) in 2018 to 25% (n = 54/217) in 2019 before COVID-19 began (Table S5). There was also no change in the prevalence of resistance to CIP or GM. Resistance to TE declined from 69 to 46% (aPR: 0.36; 95% CI 0.16, 0.77) and resistance to SXT declined from 67 to 48% (aPR: 0.48; 95% CI 0.23, 1.00) (Fig. 2, Table 2). Though changes in phenotypic resistance from 2019 to 2021 were not significantly different to changes from 2018 to 2019 pre-COVID-19, the ratios of aPRs remained < 1, indicating a greater reduction before vs. after COVID-19 began (Table S6). For example, the prevalence of resistance to CIP was 49% in 2018, 46% in 2019, and 30% in 2021 (Table S5).

The prevalence of MDR among 3GCR-EC declined from 86 to 70% (aPR: 0.32; 95% CI 0.13; 0.79), and the total number of drug classes to which each isolate was resistant declined from 3.9 to 3.3 (aPR: 0.83; 95% CI 0.73, 0.95) (Fig. 2, Table 2, Table S2). In sensitivity analyses, the prevalence of MDR and total drug classes remained unchanged from 2018 to 2019 pre-pandemic (Table S5). The most common phenotypic resistance profile among 3GCR-EC isolates in 2019 was AM CIP CZ CTX SXT TE, spanning five drug classes. The prevalence of this resistance profile decreased from 13% prior to the pandemic to 5% in 2021 (Table 3). The most common resistance profile after the pandemic began was AM CZ CTX (14%), including only the penicillin and cephalosporin drug classes, which was expected for these *E. coli* isolates since they were grown selectively for third-generation cephalosporin resistance (Table 3).

**Table 3:** Most frequently observed phenotypic resistance profiles of 3GCR-EC isolates (n = 153) from child fecal samples (n = 153) in 2021 after the COVID-19 pandemic ("Post") began compared to samples collected in 2019 before the pandemic ("Pre").

Rank	Pre (2019) (n = 97)		Post (2021) (n = 56)	
	Resistance	n (%)	Resistance	n (%)
1	AM CIP CZ CTX SXT TE	13 (13.4)	AM CZ CTX	11 (19.6)
2	AM CZ CTX SXT TE	12 (12.4)	AM CZ CTX SXT TE	5 (8.9)
3	AM CZ CTX	11 (11.3)	AM CIP CZ CTX SXT TE	3 (5.4)
4	AM CZ CTX SXT	6 (6.2)	AM CZ CTX SXT	3 (5.4)
5	AM CZ CTX TE	5 (5.1)	AM CZ SXT	3 (5.4)
6	AM CIP CZ CTX TE	4 (4.1)	AM CIP CZ SXT TE	2 (3.6)
7	AM FEP CIP CZ CTX GM SXT TE	4 (4.1)	AM FEP CIP CZ CTX GM TE	2 (3.6)
8	AM FEP CZ CTX SXT	4 (4.1)	AM FEP CIP CZ CTX SXT TE	2 (3.6)
9	AM CZ CTX GM SXT TE	3 (3.1)	AM FEP CZ CTX SXT	2 (3.6)
10	AM CZ CAZ CTX SXT TE	2 (2.1)	AM CIP CZ CTX GM	1 (1.8)

*AM* Ampicillin; *CAZ* Ceftazidime; *CIP* Ciprofloxacin; *CTX* Cefotaxime; *CZ* Cefazolin; *FEP* Cefepime; *GM* Gentamicin; *IPM* Imipenem; *SXT* Trimethoprim/sulfamethoxazole; *TE* Tetracycline.

We sequenced whole-genomes from a subset of 135 3GCR-EC isolates to characterize changes in genotypic resistance patterns. Dominant sequence types (STs) before the pandemic included ST155, ST394, ST10, ST131, and ST354, with 48 unique STs detected (Table S7). After the pandemic began, 30 unique STs were detected, with ST5041 and ST10 being the most dominant STs (Table S7). Clinically relevant STs such as ST117, ST38, ST617, and ST88 were detected before but not after COVID-19 began (Table S7). The average number of total ARGs per isolate declined from 9.9 in 2019 to 7.8 in 2021 (aPR: 0.79; 95% CI 0.65, 0.96) (Fig. 2, Table 2). The reduction in ARGs was significantly greater from 2019 to 2021 compared to pre-COVID-19 changes from 2018 to 2019 among 383 sequenced isolates included in sensitivity analyses (Table S6).

**Figure 3:**
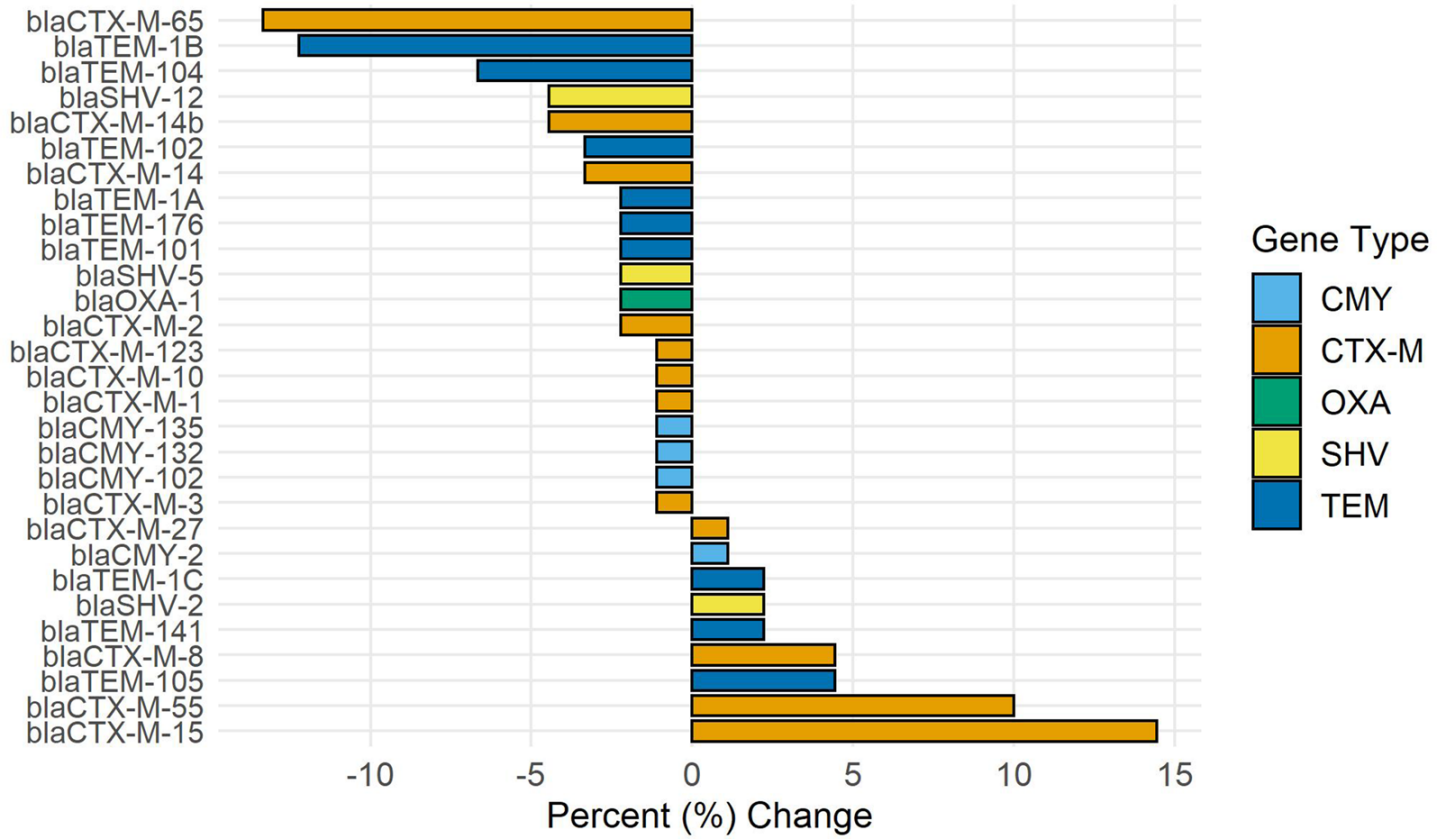
Change in the percent of sequenced 3GCR-EC isolates (N = 135) with ESBL genes in 2021 after the COVID-19 pandemic began compared to before the pandemic in 2019.

Of 383 sequenced 3GCR-EC isolates in sensitivity analyses, 98% had at least one ESBL gene, including *bla*_CTX-M_ (78%), *bla*_TEM_ (61%), *bla*_CMY_ (12%), and *bla*_SHV_ (6%), indicating ESBL production. In the main analysis, changes in ESBL or *bla* genes varied across specific ARGs, though diversity of *bla* genes seemed to decline across all types (Fig. 3). Before COVID-19, there were 12 unique *bla*_CTX-M_ genes, 8 *bla*_TEM_ genes, 4 *bla*_CMY_ genes, 2 *bla*_SHV_ genes, and 1 *bla*_OXA_ gene. After COVID-19, there were 6 unique *bla*_CTX-M_ genes, 6 *bla*_TEM_ genes, 1 *bla*_CMY_ gene, 1 *bla*_SHV_ gene, and 0 *bla*_OXA_ genes detected among sequenced isolates (Table S4). *bla*_CTX-M-55_, *bla*_CTX-M-65_, and *bla*_CTX-M-15_ were the most frequently detected ESBL genes prior to the pandemic at 26%, 22%, and 12%, respectively (Table S4). While the diversity of *bla*_CTX-M_ genes detected declined after the pandemic, the proportion of isolates with *bla*_CTX-M-55_ or *bla*_CTX-M-15_ increased to 36% and 27%, respectively (Fig. 3).

## Discussion

This study leveraged data collected before and after the COVID-19 pandemic as a natural experiment to compare antibiotic use and ARB carriage among healthy individuals in semi-rural communities of Quito, Ecuador. Overall, we observed significant declines in antibiotic use (68% reduction), community-acquired 3GCR-EC (42% reduction), MDR 3GCR-EC (18% reduction), and total ARGs (24% reduction) from 2019 to 2021 in semi-rural Quito, Ecuador.

Several factors related to population mobility and infection prevention and control measures may have contributed to the observed reduction in 3GCR-EC carriage among children. Restrictions on international travel may have limited the introduction of ESBL-E and MDR bacteria from individuals coming from subtropical regions, and restrictions in movement within communities could limit opportunities for ARB transmission^51^. International travel to low-income countries in subtropical regions—especially South Asia & Sub-Saharan Africa—is a known risk factor for ESBL-E infections, especially given antibiotic use during travel^52–54^. International travel restrictions and local quarantines in March–April 2020, the beginning of the COVID-19 pandemic, may have contributed to similar declines in ESBL-E detected in clinical isolates from community-acquired infections in France^55^.

Daycare attendance and hospital admissions are also known risk factors for ARB carriage in children^56^. Fewer children in our study site attended a daycare in 2021 compared to 2018 and 2019, preventing exposures to unhygienic or crowded daycare environments and likely reducing ARB transmission. There were also fewer children who received medical treatment in 2021 compared to pre-pandemic years. Caregivers may have decided not to seek care or schedule elective or non-urgent medical procedures for their child to avoid exposure to COVID-19 patients in hospitals and other health care centers. Telemedicine also became widely available in Ecuador during the pandemic, reducing parents’ need to visit a hospital for medical advice^57^.

Public health messaging to encourage frequent handwashing, social distancing, and use of face masks may have further reduced community transmission of infectious pathogens, including ESBL-E and other ARB^31, 32^. However, child hand washing after contact with animals decreased from 2019 to 2021 in our study site. Other measures to interrupt COVID-19 transmission like travel restrictions, curfews, and school or business closures likely played a stronger role in interrupting ARB transmission in semi-rural Ecuador. Adherence to infection prevention measures may have been higher in places with strict mandates or in areas more severely impacted by the pandemic, suggesting that these areas may have seen a greater reduction in both COVID-19 and community-acquired ARB^58–60^. This may have been the case in our study site in Quito, Ecuador, where the government enforced strict pandemic mitigation policies.

We observed 64% and 68% reductions in the proportion of children who received medical treatment and antibiotics in the last 3 months, respectively. The reduction in antibiotic use during COVID-19 is consistent with findings on antibiotic prescribing for community-acquired infections in the United States, Europe, and Australia^61–66^. While several studies have qualitatively described antibiotic use in LMIC communities during COVID-19^10–12, 67^, few have quantitatively estimated the change in community antibiotic use in LMICs since the pandemic began^68^. A single interrupted time series analysis estimated that the private health sector in India sold 216 million excess doses of antibiotics between June and September of 2020, though antibiotic sales declined from September to December of 2020 and total sales in 2020 were lower than in 2018 or 2019^68^. Despite evidence of declines in antibiotic use in our study site, community antibiotic use may rebound over time. Clinically relevant antibiotics remain unregulated and easily accessible for self-medication, which is common in poor communities in Ecuador and elsewhere^6–8^. Overuse of antibiotics among children in LMICs remains an urgent threat to children’s health, and policies restricting OTC access to antibiotics without a prescription are urgently needed to curb unnecessary or inappropriate antibiotic use^9^.

There are some limitations to this study. The parent study was not designed to test the hypothesis that there was a change in antibiotic use and resistance before vs. after the onset of the COVID-19 pandemic, increasing the potential risk of bias. We attempted to reduce bias from unmeasured confounding by only including households and children that participated in both data collection cycles before and after the start of the pandemic in 2020, allowing each household to serve as its own control. Another limitation is the use of caregiver-reported outcomes like antibiotic use and diarrhea, which increases the risk of response bias. We aimed to reduce bias in the parent study by using optimal recall periods for diarrhea (7 days) and antibiotic use (3 months), which have been shown to be reliable^69, 70^. Finally, we induced selection bias by selecting for 3GCR-EC; the observed changes in phenotypic resistance to individual antibiotics may not be representative of the prevalence of resistance among all enteric *E. coli,* let alone other Enterobacterales. Periodic surveillance or additional studies of community-acquired ARB in Ecuador and other LMICs will be necessary to more comprehensively describe the prevalence of resistance.

This analysis, which leveraged an existing dataset from a longitudinal observational study, also has several strengths. We conducted the first (to our knowledge) before and after analysis of ARB in the context of the COVID-19 pandemic in a LMIC. While the before and after analysis is clear-cut and easily interpretable, we further tested our hypotheses with a quasi-experimental sensitivity analysis via the difference in differences approach. The difference in difference method controls for longitudinal trends before COVID-19, strengthening and contextualizing the findings from the before and after analysis. Results from the main analysis were largely supported by the results of sensitivity analyses, suggesting that response bias (e.g., social-desirability bias) from repeated household visits and other secular trends did not significantly affect our findings. Finally, we included a sub-analysis of 3GCR-EC whole genomes to evaluate changes in ARGs. By adding this sequencing analysis, we were able to highlight the public health significance of 3GCR-EC carriage in this setting by identifying clinically relevant *E. coli* STs and ARGs encoding ESBL production.

Among children colonized with 3GCR-EC, several were infected with *E. coli* strains of clinical relevance. We identified several major extraintestinal pathogenic *E. coli* (ExPEC) STs including ST131, ST10, ST69, ST38, ST354, ST117, ST88, and ST617, which are responsible for a significant proportion of urinary tract and bloodstream infections globally^71^. We found a high prevalence of *bla*CTX-M and other ESBL genes among 3GCR-EC isolates, and the diversity of ESBL genes seemed to decrease after the pandemic began. CTX-M-encoding genes *bla*_CTX-M-55_, *bla*_CTX-M-65_, and *bla*_CTX-M-15_ were the most frequently detected *bla* genes in our study site in Ecuador, and are frequently found in meat products and food animals such as chickens, pigs, and cattle across Asia, North America, and Europe^72–76^. *bla*_CTX-M-15_ is also frequently detected in ExPEC, specifically the virulent and often MDR strain, ST131^77^. Previously published findings from the parent study suggest both clonal spread and horizontal gene transfer of 3GCR-EC between children, backyard chickens, and dogs in the study site, where animal waste management and handling practices were generally poor^43, 78^. Commercial food animal production is also common in this study site, and living in high-intensity production areas was identified as a risk factor for carriage of ESBL-producing 3GCR-EC among children in this study site ﻿﻿﻿(Amato et al. ^38^ In Review)﻿﻿﻿. While detection of *bla*_CTX-M-15_, *bla*_CTX-M-55_, and *bla*_CTX-M-8_ (another ESBL gene found in broiler chickens^79^) increased after COVID-19 began, detection of all other *bla*_CTX-M_ genes decreased. The decrease in diversity of ESBL genes after the pandemic began may suggest that children were exposed to fewer sources of ESBL-producing bacteria, ESBL genes, and mobile genetic elements, especially during quarantine and lockdown periods. Still, the high prevalence (31% overall) of ESBL-producing 3GCR-EC—including virulent and MDR strains—among children in Ecuadorian communities is concerning. Comprehensive strategies such as vaccine development and large-scale human and animal waste management are necessary to tackle the spread of MDR pathogenic *E. coli*^80, 81^.

## Conclusions

The COVID-19 pandemic provided an unprecedented opportunity to assess the impacts of restricted population mobility and intensive infection control and prevention measures on community transmission of resistant bacteria. Researchers with relevant data that was collected before and during the pandemic should consider conducting similar analyses to broaden our understanding of ARB transmission dynamics in various contexts. Antibiotic use and community spread of ARB during the pandemic may vary locally based on socioeconomic status, population density, proximity to urban centers, social and cultural norms, and intensity of COVID-19 control and prevention policies. Our findings suggest that in the context of severe outbreaks and strict policies to reduce COVID-19 transmission in Ecuador, community-acquired ARB decreased from 2019 to 2021. Periodic surveillance of community-acquired ARB infections will be necessary to track long-term changes in ARB transmission as pandemic-related policies continue to evolve. Knowledge, attitudes, and practices around antibiotic use may also evolve, and further improvements are needed to curb excessive and inappropriate antibiotic use. As antibiotic resistance remains an urgent threat to public health, governments should leverage public health communications systems bolstered during the pandemic to encourage and enforce antibiotic stewardship among community members as well as pharmacists, physicians, veterinarians, and food-animal producers. Improved hygiene practices (e.g., frequent handwashing, regular cleaning of surfaces, limited capacity/crowding) adapted to prevent the spread of COVID-19 should be maintained in public places, in schools/daycares, and in households to prevent ARB transmission. Finally, given the role of domestic and commercially produced food animals in community spread of ARB, public health policies and communications efforts must use a One Health approach to effectively address inappropriate antibiotic use and limit the selection for and exposures to resistant bacteria.

### Supplementary Information

Supplementary Information.

## Data Availability

Raw reads from isolates sequenced in this study are available at the NCBI Short Read Archive (SRA) under BioProject accession no. PRJNA861272. Code and de-identified data for main analysis is publicly available online at https://github.com/HeatherKAmato/AMR_Ecuador.

## Abbreviations

ARB: Antibiotic-resistant bacteria
ARG: Antibiotic resistance genes
ESBL: Extended-spectrum beta-lactamase
COVID-19: Coronavirus 2019
LMIC: Low- and middle-income countries
MDR: Multidrug-resistant
OTC: Over-the-counter
3GCR-EC: Third-generation cephalosporin-resistant *Escherichia coli*

## References

[1] Knight GM, Glover RE, McQuaid CF, Olaru ID, Gallandat K, Leclerc QJ (2021). Antimicrobial resistance and COVID-19: Intersections and implications. Elife.

[2] Bong C-L, Brasher C, Chikumba E, McDougall R, Mellin-Olsen J, Enright A (2020). The COVID-19 pandemic: Effects on low- and middle-income countries. Anesth. Analg..

[3] Alhalaseh YN, Elshabrawy HA, Erashdi M, Shahait M, Abu-Humdan AM, Al-Hussaini M (2021). Allocation of the “Already” limited medical resources amid the COVID-19 pandemic, an iterative ethical encounter including suggested solutions from a real life encounter. Front. Med..

[4] Chitungo I, Dzinamarira T, Nyazika TK, Herrera H, Musuka G, Murewanhema G (2022). Inappropriate antibiotic use in zimbabwe in the COVID-19 era: A perfect recipe for antimicrobial resistance. Antibiotics (Basel).

[5] Pokharel S, Raut S, Adhikari B (2019). Tackling antimicrobial resistance in low-income and middle-income countries. BMJ Glob. Health.

[6] Haenssgen MJ, Charoenboon N, Zanello G, Mayxay M, Reed-Tsochas F, Lubell Y (2019). Antibiotic knowledge, attitudes and practices: new insights from cross-sectional rural health behaviour surveys in low-income and middle-income South-East Asia. BMJ Open.

[7] Irawati L, Alrasheedy AA, Hassali MA, Saleem F (2019). Low-income community knowledge, attitudes and perceptions regarding antibiotics and antibiotic resistance in Jelutong District, Penang, Malaysia: A qualitative study. BMC Public Health.

[8] Do NTT, Vu HTL, Nguyen CTK, Punpuing S, Khan WA, Gyapong M (2021). Community-based antibiotic access and use in six low-income and middle-income countries: a mixed-method approach. Lancet Glob. Health.

[9] Fink G, D’Acremont V, Leslie HH, Cohen J (2020). Antibiotic exposure among children younger than 5 years in low-income and middle-income countries: a cross-sectional study of nationally representative facility-based and household-based surveys. Lancet Infect. Dis..

[10] Elsayed AA, Darwish SF, Zewail MB, Mohammed M, Saeed H, Rabea H (2021). Antibiotic misuse and compliance with infection control measures during COVID-19 pandemic in community pharmacies in Egypt. Int. J. Clin. Pract..

[11] Khojah HMJ (2022). Over-the-counter sale of antibiotics during COVID-19 outbreak by community pharmacies in Saudi Arabia: A simulated client study. BMC Health Serv. Res..

[12] Kalam MA, Shano S, Afrose S, Uddin MN, Rahman N, Jalal FA (2022). Antibiotics in the community during the COVID-19 pandemic: A qualitative study to understand users’ perspectives of antibiotic seeking and consumption behaviors in Bangladesh. Patient Prefer. Adherence.

[13] Olamijuwon, E., Konje, E., Kansiime, C., Kesby, M., Keenan, K., Neema, S. *et al*. *Antibiotic dispensing practices during COVID-19 and implications for Antimicrobial Resistance (AMR): Parallel mystery client studies in Uganda and Tanzania*. Research Square. Available from: https://www.researchsquare.com/article/rs-1276230/latest.pdf (2022). 10.1186/s13756-022-01199-4 PMC9919751 36774512

[14] Llor C, Bjerrum L (2014). Antimicrobial resistance: risk associated with antibiotic overuse and initiatives to reduce the problem. Ther. Adv. Drug Saf..

[15] King LM, Lovegrove MC, Shehab N, Tsay S, Budnitz DS, Geller AI (2021). Trends in US outpatient antibiotic prescriptions during the coronavirus disease 2019 pandemic. Clin. Infect. Dis..

[16] Rawson TM, Moore LSP, Zhu N, Ranganathan N, Skolimowska K, Gilchrist M (2020). Bacterial and fungal coinfection in individuals with coronavirus: A rapid review to support COVID-19 antimicrobial prescribing. Clin. Infect. Dis..

[17] Lai C-C, Chen S-Y, Ko W-C, Hsueh P-R (2021). Increased antimicrobial resistance during the COVID-19 pandemic. Int. J. Antimicrob. Agents.

[18] Fu Y, Yang Q, Xu M, Kong H, Chen H, Fu Y (2020). Secondary bacterial infections in critical Ill patients with coronavirus disease 2019. Open Forum Infect Dis..

[19] Emeraud C, Figueiredo S, Bonnin RA, Khecharem M, Ouzani S, Leblanc P-E (2021). Outbreak of CTX-M-15 extended-spectrum β-lactamase-producing Klebsiella pneumoniae ST394 in a french intensive care unit dedicated to COVID-19. Pathogens.

[20] Amarsy R, Trystram D, Cambau E, Monteil C, Fournier S, Oliary J (2022). Surging bloodstream infections and antimicrobial resistance during the first wave of COVID–19: A study in a large multihospital institution in the Paris region. Int. J. Infect. Dis..

[21] Vijay S, Bansal N, Rao BK, Veeraraghavan B, Rodrigues C, Wattal C (2021). Secondary infections in hospitalized COVID-19 patients: Indian experience. Infect. Drug Resist..

[22] Flokas ME, Karanika S, Alevizakos M, Mylonakis E (2017). Prevalence of ESBL-producing enterobacteriaceae in pediatric bloodstream infections: A systematic review and meta-analysis. PLoS ONE.

[23] MacVane SH, Tuttle LO, Nicolau DP (2014). Impact of extended-spectrum β-lactamase-producing organisms on clinical and economic outcomes in patients with urinary tract infection. J. Hosp. Med..

[24] Ray S, Anand D, Purwar S, Samanta A, Upadhye KV, Gupta P (2018). Association of high mortality with extended–spectrum β-lactamase (ESBL) positive cultures in community acquired infections. J. Crit. Care.

[25] Cosgrove SE, Kaye KS, Eliopoulous GM, Carmeli Y (2002). Health and economic outcomes of the emergence of third-generation cephalosporin resistance in Enterobacter species. Arch. Intern. Med..

[26] Bezabih YM, Sabiiti W, Alamneh E, Bezabih A, Peterson GM, Bezabhe WM (2021). The global prevalence and trend of human intestinal carriage of ESBL-producing *Escherichia coli* in the community. J. Antimicrob. Chemother..

[27] Nadimpalli M, Delarocque-Astagneau E, Love DC, Price LB, Huynh B-T, Collard J-M (2018). Combating global antibiotic resistance: Emerging one health concerns in lower- and middle-income countries. Clin. Infect. Dis..

[28] Collignon P, Beggs JJ, Walsh TR, Gandra S, Laxminarayan R (2018). Anthropological and socioeconomic factors contributing to global antimicrobial resistance: a univariate and multivariable analysis. Lancet Planet. Health..

[29] Graham DW, Collignon P, Davies J, Larsson DGJ, Snape J (2014). Underappreciated role of regionally poor water quality on globally increasing antibiotic resistance. Environ. Sci. Technol..

[30] Collignon P, Beggs JJ (2020). CON: COVID-19 will not result in increased antimicrobial resistance prevalence. JAC Antimicrob. Resist..

[31] Nadimpalli ML, Stewart JR, Pierce E, Pisanic N, Love DC, Hall D (2018). Face mask use and persistence of livestock-associated Staphylococcus aureus nasal carriage among industrial hog operation workers and household contacts, USA. Environ. Health Perspect..

[32] Kardaś-Słoma L, Yazdanpanah Y, Perozziello A, Zahar J-R, Lescure F-X, Cousien A (2020). Hand hygiene improvement or antibiotic restriction to control the household transmission of extended-spectrum β-lactamase-producing *Escherichia coli*: A mathematical modelling study. Antimicrob. Resist. Infect. Control..

[33] Cevallos-Valdiviezo H, Vergara-Montesdeoca A, Zambrano-Zambrano G (2021). Measuring the impact of the COVID-19 outbreak in Ecuador using preliminary estimates of excess mortality, March 17-October 22. Int. J. Infect. Dis..

[34] Hale T, Angrist N, Goldszmidt R, Kira B, Petherick A, Phillips T (2021). A global panel database of pandemic policies (Oxford COVID-19 Government Response Tracker). Nat. Hum. Behav..

[35] Ecuador lifts indoor and outdoor mask mandates. Reuters. Accessed 29 Apr 2022 [cited 2022 Jul 18]; Available from: https://www.reuters.com/world/americas/ecuador-lifts-indoor-outdoor-mask-mandates-2022-04-29/

[36] Ecuador’s largest city eases quarantine as COVID-19 deaths decline. Reuters. Accessed 21 May 2020 [cited 2022 Jul 18]; Available from: https://www.reuters.com/article/us-health-coronavirus-ecuador-guayaquil-idUSKBN22X00C

[37] Crisis. Ecuador: Officials announce new COVID-19 measures, including curfew and travel restrictions /update 16. Crisis24. 2022 [cited 2022 Jul 18]. Available from: https://crisis24.garda.com/alerts/2020/12/ecuador-officials-announce-new-covid-19-measures-including-curfew-and-travel-restrictions-update-16

[38] Amato, H.K., Loayza, F., Salinas, L., Paredes, D., García, D., Sarzosa, S., Saraiva, C., Johnson, T., Pickering, A.J., Riley, L.W. and Trueba, G., 2022. Risk factors for extended-spectrum beta-lactamase (ESBL) producing E. coli carriage among children in a food animal producing region of Quito, Ecuador. medRxiv, pp.2022–11. 10.1371/journal.pmed.1004299 PMC10621961 37831716

[39] CLSI-Clinical, Institute LS. Performance standards for antimicrobial susceptibility testing, Twenty-Fourth Informational Supplement, CLSI document M100-S24. CLSI Wayne; (2014).

[40] Bolger AM, Lohse M, Usadel B (2014). Trimmomatic: A flexible trimmer for Illumina sequence data. Bioinformatics.

[41] Larsen MV, Cosentino S, Rasmussen S, Friis C, Hasman H, Marvig RL (2012). Multilocus sequence typing of total-genome-sequenced bacteria. J. Clin. Microbiol..

[42] Alikhan N-F, Zhou Z, Sergeant MJ, Achtman M (2018). A genomic overview of the population structure of Salmonella. PLoS Genet..

[43] Salinas L, Loayza F, Cárdenas P, Saraiva C, Johnson TJ, Amato H (2021). Environmental spread of extended spectrum beta-lactamase (ESBL) producing *Escherichia coli* and ESBL genes among children and domestic animals in Ecuador. Environ. Health Perspect..

[44] Lee DS, Choe H-S, Kim HY, Yoo JM, Bae WJ, Cho YH (2016). Role of age and sex in determining antibiotic resistance in febrile urinary tract infections. Int. J. Infect. Dis..

[45] R Core Team. R: A Language and Environment for Statistical Computing. Vienna, Austria: R Foundation for Statistical Computing. Available from: https://www.R-project.org (2020).

[46] Wickham, H., François, R., Henry, L., Müller, K., RStudio. dplyr: A Grammar of Data Manipulation. Available from: https://CRAN.R-project.org/package=dplyr (2021).

[47] Heinzen, E., Sinnwell, J., Atkinson, E., Gunderson, T., Dougherty, G., Votruba, P. *et al*. arsenal: An Arsenal of “R” Functions for Large-Scale Statistical Summaries. Available from: https://CRAN.R-project.org/package=arsenal (2020).

[48] Halekoh U, Højsgaard S, Yan J (2006). The R package geepack for generalized estimating equations. J. Stat. Softw..

[49] Wickham, H., Chang, W., Henry, L., Pedersen, T.L., Takahashi, K., Wilke, C. *et al*. ggplot2: Create Elegant Data Visualisations Using the Grammar of Graphics. Available from: https://CRAN.R-project.org/package=ggplot2 (2021).

[50] Wing C, Simon K, Bello-Gomez RA (2018). Designing difference in difference studies: Best practices for public health policy research. Annu. Rev. Public Health.

[51] MacPherson DW, Gushulak BD, Baine WB, Bala S, Gubbins PO, Holtom P (2009). Population mobility, globalization, and antimicrobial drug resistance. Emerg. Infect. Dis..

[52] Arcilla MS, van Hattem JM, Haverkate MR, Bootsma MCJ, van Genderen PJJ, Goorhuis A (2017). Import and spread of extended-spectrum β-lactamase-producing Enterobacteriaceae by international travellers (COMBAT study): A prospective, multicentr cohort study. Lancet Infect. Dis..

[53] Ruppé E, Armand-Lefèvre L, Estellat C, Consigny P-H, El Mniai A, Boussadia Y (2015). High rate of acquisition but short duration of carriage of multidrug-resistant enterobacteriaceae after travel to the tropics. Clin. Infect. Dis..

[54] Woerther P-L, Andremont A, Kantele A (2017). Travel-acquired ESBL-producing Enterobacteriaceae: Impact of colonization at individual and community level. J. Travel Med..

[55] Lemenand O, Coeffic T, Thibaut S, Colomb Cotinat M, Caillon J, Birgand G (2021). Decreasing proportion of extended-spectrum beta-lactamase among *E. coli* infections during the COVID-19 pandemic in France. J. Infect..

[56] Chan YQ, Chen K, Chua GT, Wu P, Tung KTS, Tsang HW (2022). Risk factors for carriage of antimicrobial-resistant bacteria in community dwelling-children in the Asia-Pacific region: A systematic review and meta-analysis. JAC Antimicrob. Resist..

[57] View of the role of telemedicine on Ecuador during the COVID-19 crisis: A perspective from a volunteer physician. [cited 2022 Jul 30]. Available from: https://www.ijms.info/IJMS/article/view/547/353

[58] Dwipayanti NMU, Lubis DS, Harjana NPA (2021). Public perception and hand hygiene behavior during COVID-19 pandemic in Indonesia. Front. Public Health.

[59] Huang J, Fisher BT, Tam V, Wang Z, Song L, Shi J (2022). The Effectiveness Of Government Masking Mandates On COVID-19 County-Level Case Incidence Across The United States, 2020. Health Aff..

[60] Adjodah D, Dinakar K, Chinazzi M, Fraiberger SP, Pentland A, Bates S (2021). Association between COVID-19 outcomes and mask mandates, adherence, and attitudes. PLoS ONE.

[61] Gillies MB, Burgner DP, Ivancic L, Nassar N, Miller JE, Sullivan SG (2022). Changes in antibiotic prescribing following COVID-19 restrictions: Lessons for post-pandemic antibiotic stewardship. Br J. Clin. Pharmacol..

[62] Rezel-Potts E, L’Esperance V, Gulliford MC (2021). Antimicrobial stewardship in the UK during the COVID-19 pandemic: a population-based cohort study and interrupted time-series analysis. Br J. Gen. Pract..

[63] Peñalva G, Benavente RS, Pérez-Moreno MA, Pérez-Pacheco MD, Pérez-Milena A, Murcia J (2021). Effect of the coronavirus disease 2019 pandemic on antibiotic use in primary care. Clin. Microbiol. Infect..

[64] Buehrle DJ, Nguyen MH, Wagener MM, Clancy CJ (2020). Impact of the coronavirus disease 2019 pandemic on outpatient antibiotic prescriptions in the United States. Open Forum. Infect Dis..

[65] van de Pol AC, Boeijen JA, Venekamp RP, Platteel T, Damoiseaux RAMJ, Kortekaas MF (2021). Impact of the COVID-19 pandemic on antibiotic prescribing for common infections in The Netherlands: A primary care-based observational cohort study. Antibiotics (Basel)..

[66] Armitage R, Nellums LB (2021). Antibiotic prescribing in general practice during COVID-19. Lancet Infect. Dis..

[67] Opanga SA, Rizvi N, Wamaitha A, Sefah IA, Godman B (2021). Availability of medicines in community pharmacy to manage patients with COVID-19 in Kenya; pilot study and implications. Sch. Acad. J. Pharm..

[68] Sulis G, Batomen B, Kotwani A, Pai M, Gandra S (2021). Sales of antibiotics and hydroxychloroquine in India during the COVID-19 epidemic: An interrupted time series analysis. PLoS Med..

[69] Arnold BF, Galiani S, Ram PK, Hubbard AE, Briceño B, Gertler PJ (2013). Optimal recall period for caregiver-reported illness in risk factor and intervention studies: A multicountry study. Am. J. Epidemiol..

[70] Demoré B, Le Govic D, Thilly N, Boivin J-M, Pulcini C (2017). Reliability of self-reported recent antibiotic use among the general population: A cross-sectional study. Clin. Microbiol. Infect..

[71] Manges AR, Geum HM, Guo A, Edens TJ, Fibke CD, Pitout JDD (2019). Global extraintestinal pathogenic *Escherichia coli* (ExPEC) lineages. Clin. Microbiol. Rev..

[72] Leão C, Clemente L, Moura L, Seyfarth AM, Hansen IM, Hendriksen RS (2021). Emergence and clonal spread of CTX-M-65-producing Escherichia coli from retail meat in Portugal. Front. Microbiol..

[73] Zheng H, Zeng Z, Chen S, Liu Y, Yao Q, Deng Y (2012). Prevalence and characterisation of CTX-M β-lactamases amongst *Escherichia coli* isolates from healthy food animals in China. Int. J. Antimicrob. Agents.

[74] Park H, Kim J, Ryu S, Jeon B (2019). Predominance of blaCTX-M-65 and blaCTX-M-55 in extended-spectrum β-lactamase-producing *Escherichia coli* from raw retail chicken in South Korea. J. Glob. Antimicrob. Resist..

[75] Ho, Chow, Lai, Lo. Extensive dissemination of CTX-M-producing *Escherichia coli* with multidrug resistance to “critically important” antibiotics among food animals in Hong Kong, 2008–10. J. At. Mol. Phys. Available from: https://academic.oup.com/jac/article-abstract/66/4/765/725063 10.1093/jac/dkq539 21393133

[76] Cormier A, Zhang PLC, Chalmers G, Weese JS, Deckert A, Mulvey M (2019). Diversity of CTX-M-positive *Escherichia coli* recovered from animals in Canada. Vet. Microbiol..

[77] Mathers AJ, Peirano G, Pitout JDD (2015). *Escherichia coli* ST131: The quintessential example of an international Multiresistant high-risk clone. Adv. Appl. Microbiol..

[78] Salinas L, Cárdenas P, Johnson TJ, Vasco K, Graham J, Trueba G (2019). Diverse commensal *Escherichia coli* clones and plasmids disseminate antimicrobial resistance genes in domestic animals and children in a semirural community in Ecuador. mSphere.

[79] Gundran RS, Cardenio PA, Villanueva MA, Sison FB, Benigno CC, Kreausukon K (2019). Prevalence and distribution of blaCTX-M, blaSHV, blaTEM genes in extended- spectrum β- lactamase- producing *E. coli* isolates from broiler farms in the Philippines. BMC Vet. Res..

[80] Poolman JT, Wacker M (2016). Extraintestinal pathogenic *Escherichia coli*, a common human pathogen: Challenges for vaccine development and progress in the field. J. Infect. Dis..

[81] Musoke D, Namata C, Lubega GB, Niyongabo F, Gonza J, Chidziwisano K (2021). The role of Environmental Health in preventing antimicrobial resistance in low- and middle-income countries. Environ. Health Prev. Med..

