# Supplementary material for: Leveraging the COVID-19 pandemic as a natural experiment to assess changes in antibiotic use and antibiotic-resistant E. coli carriage in semi-rural Ecuador
Source: Sci Rep. 2023 Sep 8;13:14854. doi: 10.1038/s41598-023-39532-5 (PMC10491794; doi:10.1038/s41598-023-39532-5)
Supplement: Supplementary file 1 — Supplementary Information. [file 41598_2023_39532_MOESM1_ESM.docx]

## Supplemental Materials

#### Figure S1. Study timeline and methodology for main analysis and sensitivity analysis.

###
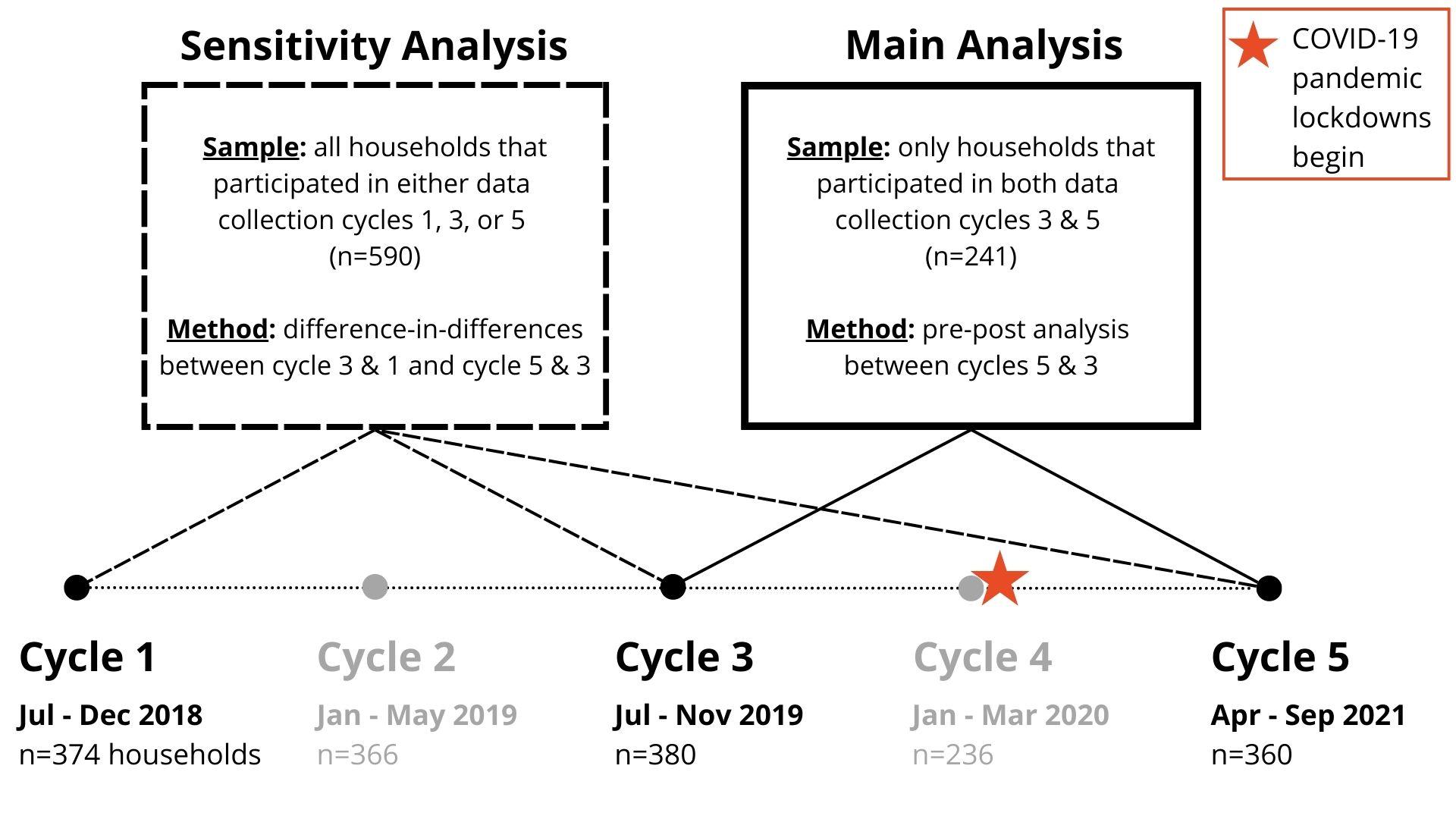


#### Table S1. Caregiver knowledge, attitudes, and practices around antibiotic use before and after the beginning of the COVID-19 pandemic (data corresponds to Figure 1 in main text).

|  | **Pre (2019)**  *n (%)* | **Post (2021)**  *n (%)* | **Total**  *n (%)* |
| --- | --- | --- | --- |
| *Total households* | 241 (100) | 241 (100) | 482 (100) |
| *Caregiver believes antibiotics kill bacteria* |  |  |  |
| Yes | 91 (37.8) | 107 (44.4) | 198 (41.1) |
| No | 63 (26.1) | 67 (27.8) | 130 (27.0) |
| Don’t know | 87 (36.1) | 67 (27.8) | 154 (32.0) |
| *Caregiver believes antibiotics kill viruses* |  |  |  |
| Yes | 84 (34.9) | 78 (32.4) | 162 (33.6) |
| No | 66 (27.4) | 91 (37.8) | 157 (32.6) |
| Don’t know | 91 (37.8) | 72 (29.9) | 163 (33.8) |
| *Caregiver gives their child antibiotics for a sore throat* |  |  |  |
| Never | 47 (19.5) | 51 (21.2) | 98 (20.3) |
| Always | 103 (42.7) | 34 (14.1) | 137 (28.4) |
| Sometimes | 90 (37.3) | 151 (62.7) | 241 (50.0) |
| Don’t know | 1 (0.4) | 5 (2.1) | 6 (1.2) |
| *Caregiver believes giving their child antibiotics for a cold will make them better* | | |  |
| Never | 61 (25.3) | 44 (18.3) | 105 (21.8) |
| Always | 107 (44.4) | 65 (27.0) | 172 (35.7) |
| Sometimes | 70 (29.0) | 122 (50.6) | 192 (39.8) |
| Don’t know | 3 (1.2) | 10 (4.1) | 13 (2.7) |
| *Caregiver expects the doctor to prescribe antibiotics if their child has a bad cold* | | |  |
| Never | 50 (20.7) | 24 (10.0) | 74 (15.4) |
| Always | 121 (50.2) | 86 (35.7) | 207 (42.9) |
| Sometimes | 69 (28.6) | 117 (48.5) | 186 (38.6) |
| Don’t know | 1 (0.4) | 14 (5.8) | 15 (3.1) |
| *Caregiver believes it’s okay to take antibiotics when feeling sick* |  |  |  |
| Never | 43 (17.8) | 23 (9.5) | 66 (13.7) |
| Always | 77 (32.0) | 43 (17.8) | 120 (24.9) |
| Sometimes | 117 (48.5) | 161 (66.8) | 278 (57.7) |
| Don’t know | 4 (1.7) | 14 (5.8) | 18 (3.7) |
| *Antibiotics are available to buy at the pharmacy* |  |  |  |
| Never | 141 (58.5) | 88 (36.5) | 229 (47.5) |
| Always | 39 (16.2) | 27 (11.2) | 66 (13.7) |
| Sometimes | 54 (22.4) | 100 (41.5) | 154 (32.0) |
| Don’t know | 7 (2.9) | 26 (10.8) | 33 (6.8) |
| *Caregiver believes that most of their friends think they should give their child antibiotics for a cold* | | | |
| Never | 38 (15.8) | 25 (10.4) | 63 (13.1) |
| Always | 70 (29.0) | 16 (6.7) | 86 (17.9) |
| Sometimes | 83 (34.4) | 126 (52.5) | 209 (43.5) |
| Don’t know | 50 (20.7) | 73 (30.4) | 123 (25.6) |
| Missing | 0 | 1 | 1 |
| *Caregiver believes that most of their friends think they should give their child antibiotics for diarrhea* | | | |
| Never | 43 (17.8) | 29 (12.0) | 72 (14.9) |
| Always | 72 (29.9) | 17 (7.1) | 89 (18.5) |
| Sometimes | 74 (30.7) | 94 (39.0) | 168 (34.9) |
| Don’t know | 52 (21.6) | 101 (41.9) | 153 (31.7) |
| *Caregiver believes that most of their friends think they should give their child antibiotics for skin rashes* | | | |
| Never | 52 (21.6) | 23 (9.5) | 75 (15.6) |
| Always | 67 (27.8) | 12 (5.0) | 79 (16.4) |
| Sometimes | 70 (29.0) | 94 (39.0) | 164 (34.0) |
| Don’t know | 52 (21.6) | 112 (46.5) | 164 (34.0) |
| *Caregiver believes that most of their friends buy their child antibiotics without a prescription* | | | |
| Never | 47 (19.5) | 24 (10.0) | 71 (14.7) |
| Always | 57 (23.7) | 9 (3.7) | 66 (13.7) |
| Sometimes | 88 (36.5) | 131 (54.4) | 219 (45.4) |
| Don’t know | 49 (20.3) | 77 (32.0) | 126 (26.1) |

###

#### Table S2. Caregiver-reported hygiene practices, child illness, antibiotic use, carriage of third-generation cephalosporin-resistant E. coli (3GCR-EC), and antibiotic resistance in 3GCR-EC isolated from child fecal samples collected before (Pre) and after (Post) the beginning of the COVID-19 pandemic.

|  | **Pre (2019)**  *n (%)* | **Post (2021)**  *n (%)* | **Total**  *n (%)* |
| --- | --- | --- | --- |
| *Total* | 241 (100) | 241 (100) | 482 (100) |
| *Child had contact with pets in last 3 months* | 177 (73.4%) | 178 (73.9%) | 355 (73.7%) |
| *Child had contact with livestock in last 3 months* | 88 (36.5%) | 119 (49.4%) | 207 (42.9%) |
| *Child washes hands after contact with animals sometimes/always (vs. rarely/never)* | 170 (70.5) | 101 (41.9) | 271 (56.2) |
| *Child attends daycare* | 28 (11.7%) | 9 (3.8%) | 37 (7.7%) |
| Missing | 0 | 2 | 2 |
| *Child had diarrhea in the last 7 days* | 28 (11.6) | 9 (3.7) | 37 (7.7) |
| Missing | 1 | 1 | 2 |
| *Child received medical treatment in last 3 months* | 56 (23.2) | 20 (8.3) | 76 (15.8) |
| *Child took an antibiotic in last 3 months* | 41 (17.0) | 13 (5.4) | 54 (11.2) |
| Missing | 0 | 1 | 1 |
| *3GCR-EC carriage* | 97 (40.2) | 56 (23.2) | 153 (31.2) |
| Missing | 4 | 4 | 8 |
| *Phenotypic resistance to antibiotics among*  *3GCR-EC isolates* | 97 (100) | 56 (100) | 153 (100) |
| AM | 96 (99.0) | 52 (92.9) | 148 (96.7) |
| CTX | 90 (92.8) | 44 (78.6) | 134 (87.6) |
| CZ | 96 (99.0) | 53 (94.6) | 149 (97.4) |
| CAZ | 23 (23.7) | 7 (12.5) | 30 (19.6) |
| FEP | 26 (26.8) | 14 (25.0) | 40 (26.1) |
| IPM | 1 (1.0) | 1 (1.8) | 2 (1.3) |
| SXT | 65 (67.0) | 27 (48.2) | 92 (60.1) |
| CIP | 45 (46.4) | 17 (30.4) | 62 (40.5) |
| GM | 17 (17.5) | 6 (10.7) | 23 (15.0) |
| TE | 67 (69.1) | 26 (46.4) | 93 (60.8) |
| MDR (3+ classes) | 82 (84.5) | 39 (69.6) | 121 (79.1) |
| Total classes; mean (SD) | 3.85 (1.22) | 3.25 (1.39) | 3.63 (1.31) |
| *Abundance of ARGs among sequenced*  *3GCR-EC isolates (n=135)* |  |  |  |
| Total ARGs; mean (SD) | 9.92 (4.64) | 7.76 (4.34) | 9.20 (4.64) |

#### Table S3. Changes in hygiene practices and risk factors for antibiotic resistance carriage before and after the COVID-19 pandemic began.

|  | **Pre (2019)**  *n (%)* | **Post (2021)**  *n (%)* | **Total**  *n (%)* |
| --- | --- | --- | --- |
| *Total* | 241 (100) | 241 (100) | 482 (100) |
| *Child had contact with pets in last 3 months* | 177 (73.4%) | 178 (73.9%) | 355 (73.7%) |
| *Child had contact with livestock in last 3 months* | 88 (36.5%) | 119 (49.4%) | 207 (42.9%) |
| *Child washes hands after contact with animals*  *sometimes/always (vs. rarely/never)* | 170 (70.5) | 101 (41.9) | 271 (56.2) |
| *Child attends daycare* | 28 (11.7%) | 9 (3.8%) | 37 (7.7%) |
| Missing | 0 | 2 | 2 |

####

#### Table S4. Detection of ESBL genes among sequenced 3GCR-EC isolates from child fecal samples collected before and after the COVID-19 pandemic began.

|  |  | No. 3GCR-EC Isolates (%) | |
| --- | --- | --- | --- |
| **Group** | **Gene** | **Pre (2019)**  (n=90) | **Post (2021)**  (n=45) |
| CMY | *bla*_CMY-102_ | 1 (1.11) | 0 (0) |
|  | *bla*_CMY-132_ | 1 (1.11) | 0 (0) |
|  | *bla*_CMY-135_ | 1 (1.11) | 0 (0) |
|  | *bla*_CMY-2_ | 7 (7.78) | 4 (8.89) |
| CTX-M | *bla*_CTX-M-1_ | 1 (1.11) | 0 (0) |
|  | *bla*_CTX-M-10_ | 1 (1.11) | 0 (0) |
|  | *bla*_CTX-M-123_ | 1 (1.11) | 0 (0) |
|  | *bla*_CTX-M-14_ | 3 (3.33) | 0 (0) |
|  | *bla*_CTX-M-14b_ | 4 (4.44) | 0 (0) |
|  | *bla*_CTX-M-15_ | 11 (12.22) | 12 (26.67) |
|  | *bla*_CTX-M-2_ | 2 (2.22) | 0 (0) |
|  | *bla*_CTX-M-27_ | 3 (3.33) | 2 (4.44) |
|  | *bla*_CTX-M-3_ | 3 (3.33) | 1 (2.22) |
|  | *bla*_CTX-M-55_ | 23 (25.56) | 16 (35.56) |
|  | *bla*_CTX-M-65_ | 20 (22.22) | 4 (8.89) |
|  | *bla*_CTX-M-8_ | 2 (2.22) | 3 (6.67) |
| OXA | *bla*_OXA-1_ | 2 (2.22) | 0 (0) |
| SHV | *bla*_SHV-12_ | 4 (4.44) | 0 (0) |
|  | *bla*_SHV-5_ | 2 (2.22) | 0 (0) |
|  | *bla*_SHV-2_ | 0 (0) | 1 (2.22) |
| TEM | *bla*_TEM-101_ | 2 (2.22) | 0 (0) |
|  | *bla*_TEM-102_ | 7 (7.78) | 2 (4.44) |
|  | *bla*_TEM-104_ | 12 (13.33) | 3 (6.67) |
|  | *bla*_TEM-105_ | 10 (11.11) | 7 (15.56) |
|  | *bla*_TEM-141_ | 22 (24.44) | 12 (26.67) |
|  | bla_TEM-176_ | 2 (2.22) | 0 (0) |
|  | *bla*_TEM-1A_ | 2 (2.22) | 0 (0) |
|  | *bla*_TEM-1B_ | 27 (30) | 8 (17.78) |
|  | *bla*_TEM-1C_ | 0 (0) | 1 (2.22) |

###

#### Table S5. Data distribution from sensitivity analyses comparing changes from 2019 to 2021 (pre- vs. post-COVID-19 began) to changes from 2018 to 2019 (both pre-COVID-19), including all households.

|  | **Pre (2018)**  *n (%)* | **Pre (2019)**  *n (%)* | **Post (2021)**  *n (%)* |
| --- | --- | --- | --- |
| *Total households/children* | 374 (100) | 380 (100) | 360 (100) |
| *Caregiver believes antibiotics kill bacteria* |  |  |  |
| Yes | 138 (37.1%) | 138 (36.3%) | 147 (40.8%) |
| No | 75 (20.2%) | 108 (28.4%) | 119 (33.1%) |
| Don’t know | 159 (42.7%) | 134 (35.3%) | 94 (26.1%) |
| *Caregiver believes antibiotics kill viruses* |  |  |  |
| Yes | 129 (34.6%) | 129 (33.9%) | 131 (36.4%) |
| No | 93 (24.9%) | 106 (27.9%) | 130 (36.1%) |
| Don’t know | 151 (40.5%) | 145 (38.2%) | 99 (27.5%) |
| *Caregiver gives their child antibiotics for a sore throat* |  |  |  |
| Never | 104 (28.3%) | 66 (17.4%) | 85 (23.6%) |
| Always | 117 (31.9%) | 156 (41.1%) | 60 (16.7%) |
| Sometimes | 138 (37.6%) | 156 (41.1%) | 207 (57.5%) |
| Don’t know | 8 (2.2%) | 2 (0.5%) | 8 (2.2%) |
| Missing | 7 | 0 | 0 |
| *Caregiver believes giving their child antibiotics for a cold will make them better* | | |  |
| Never | 93 (25.1%) | 96 (25.3%) | 71 (19.7%) |
| Always | 144 (38.9%) | 172 (45.3%) | 101 (28.1%) |
| Sometimes | 102 (27.6%) | 108 (28.4%) | 175 (48.6%) |
| Don’t know | 31 (8.4%) | 4 (1.1%) | 13 (3.6%) |
| Missing | 4 | 0 | 0 |
| *Caregiver expects the doctor to prescribe antibiotics if their child has a bad cold* | | |  |
| Never | 77 (20.6%) | 76 (20.0%) | 36 (10.0%) |
| Always | 178 (47.7%) | 183 (48.2%) | 130 (36.1%) |
| Sometimes | 109 (29.2%) | 119 (31.3%) | 179 (49.7%) |
| Don’t know | 9 (2.4%) | 2 (0.5%) | 15 (4.2%) |
| Missing | 1 | 0 | 0 |
| *Caregiver believes it’s okay to take antibiotics when feeling sick* |  |  |  |
| Never | 57 (15.3%) | 66 (17.4%) | 32 (8.9%) |
| Always | 117 (31.5%) | 117 (30.8%) | 71 (19.7%) |
| Sometimes | 185 (49.7%) | 190 (50.0%) | 237 (65.8%) |
| Don’t know | 13 (3.5%) | 7 (1.8%) | 20 (5.6%) |
| Missing | 2 | 0 | 0 |
| *Antibiotics are available to buy at the pharmacy* |  |  |  |
| Never | 198 (53.2%) | 212 (55.8%) | 120 (33.3%) |
| Always | 82 (22.0%) | 59 (15.5%) | 44 (12.2%) |
| Sometimes | 79 (21.2%) | 99 (26.1%) | 157 (43.6%) |
| Don’t know | 13 (3.5%) | 10 (2.6%) | 39 (10.8%) |
| Missing | 2 | 0 | 0 |
| *Caregiver believes that most of their friends think they should give their child antibiotics for a cold* | | | |
| Never | 83 (22.5%) | 67 (17.6%) | 37 (10.3%) |
| Always | 90 (24.4%) | 104 (27.4%) | 22 (6.1%) |
| Sometimes | 97 (26.3%) | 133 (35.0%) | 190 (52.9%) |
| Don’t know | 99 (26.8%) | 76 (20.0%) | 110 (30.6%) |
| Missing | 5 | 0 | 1 |
| *Caregiver believes that most of their friends think they should give their child antibiotics for diarrhea* | | | |
| Never | 78 (21.2%) | 70 (18.4%) | 42 (11.7%) |
| Always | 89 (24.2%) | 106 (27.9%) | 24 (6.7%) |
| Sometimes | 98 (26.6%) | 118 (31.1%) | 143 (39.7%) |
| Don’t know | 103 (28.0%) | 86 (22.6%) | 151 (41.9%) |
| Missing | 6 | 0 | 0 |
| *Caregiver believes that most of their friends think they should give their child antibiotics for skin rashes* | | | |
| Never | 84 (22.8%) | 83 (21.8%) | 33 (9.2%) |
| Always | 84 (22.8%) | 102 (26.8%) | 16 (4.4%) |
| Sometimes | 98 (26.6%) | 106 (27.9%) | 137 (38.1%) |
| Don’t know | 103 (27.9%) | 89 (23.4%) | 174 (48.3%) |
| Missing | 5 | 0 | 0 |
| *Caregiver believes that most of their friends buy their child antibiotics without a prescription* | | | |
| Never | 76 (20.7%) | 71 (18.7%) | 31 (8.6%) |
| Always | 87 (23.6%) | 96 (25.3%) | 17 (4.7%) |
| Sometimes | 93 (25.3%) | 133 (35.0%) | 188 (52.2%) |
| Don’t know | 112 (30.4%) | 80 (21.1%) | 124 (34.4%) |
| Missing | 6 | 0 | 0 |
| *Child had contact with pets in last 3 months* | 240 (64.2%) | 266 (70.0%) | 244 (67.8%) |
| *Child had contact with livestock in last 3 months* | 124 (33.2%) | 133 (35.0%) | 152 (42.2%) |
| *Child sometimes/always washes hands after contact with animals* | 186 (56.0%) | 254 (66.8%) | 159 (44.2%) |
| Missing | 42 | 0 | 0 |
| *Child attends daycare* | 52 (13.9%) | 73 (19.2%) | 52 (14.5%) |
| Missing | 0 | 0 | 2 |
| *Child had diarrhea in last 7 days* | 76 (20.4%) | 46 (12.1%) | 15 (4.2%) |
| Missing | 2 | 1 | 2 |
| *Child received medical treatment in last 3 months* | 117 (31.5%) | 88 (23.2%) | 29 (8.1%) |
| Missing | 3 | 0 | 0 |
| *Child took an antibiotic in last 3 months* | 97 (26.1%) | 64 (16.9%) | 20 (5.6%) |
| Missing | 2 | 2 | 1 |
| *Total observations** | 456 (100) | 436 (100) | 350 (100) |
| *3GCR-EC carriage* | 273 (59.9%) | 217 (49.7%) | 71 (20.3%) |
| Missing | 7 | 18 | 8 |
| *Phenotypic resistance to antibiotics among 3GCR-EC isolates* | 273 (100) | 217 (100) | 71 (100) |
| AM | 274 (99.3%) | 215 (99.1%) | 67 (94.4%) |
| CTX | 255 (92.4%) | 199 (91.7%) | 58 (81.7%) |
| CZ | 276 (100.0%) | 215 (99.1%) | 68 (95.8%) |
| CAZ | 72 (26.1%) | 48 (22.1%) | 11 (15.5%) |
| FEP | 121 (43.8%) | 54 (24.9%) | 20 (28.2%) |
| IPM | 2 (0.7%) | 1 (0.5%) | 1 (1.4%) |
| SXT | 185 (67.0%) | 144 (66.4%) | 39 (54.9%) |
| CIP | 136 (49.3%) | 100 (46.1%) | 21 (29.6%) |
| GM | 47 (17.0%) | 32 (14.7%) | 8 (11.3%) |
| TE | 201 (72.8%) | 152 (70.0%) | 40 (56.3%) |
| MDR (3+ classes) | 234 (85.7%) | 188 (86.6%) | 53 (74.6%) |
| Total classes; mean (SD) | 4.07 (1.26) | 3.96 (1.18) | 3.44 (1.34) |
| *Abundance of ARGs among sequenced 3GCR-EC isolates (n=383)* |  |  |  |
| Total ARGs; mean (SD) | 9.80 (4.41) | 9.87 (4.47) | 8.16 (4.55) |

*Includes multiple isolates per child fecal sample in cycles 1 and 3.

#### Table S6. Sensitivity analyses using a difference-in-differences approach to compare changes in cycle 5 vs. 3 (pre- vs. post-COVID-19) to changes in cycle 3 vs. 1 (both pre-COVID-19).

| **Category** | **Outcome Variable** | **N** | **RaPR* (95% CI)** | ***P-*value** |
| --- | --- | --- | --- | --- |
| *Caregiver knowledge about antibiotics* | Caregiver believes antibiotics kill bacteria  (ref = yes) | 1455 | 1.26 (0.79, 2.03) | 0.3383 |
|  | Caregiver believes antibiotics kill viruses  (ref = no) | 1456 | 0.86 (0.54, 1.37) | 0.5232 |
| *Caregiver attitudes about antibiotic use*  (ref = sometimes) | Caregiver gives their child antibiotics for a  sore throat | 1450 | 0.47 (0.30, 0.72) | 0.006 |
|  | Caregiver believes giving their child antibiotics for a cold will make them better | 1453 | 0.42 (0.26, 0.65) | 0.0001 |
|  | Caregiver expects the doctor to prescribe antibiotics if their child has a bad cold | 1456 | 0.50 (0.32, 0.78) | 0.0021 |
|  | Caregiver believes it’s okay to take antibiotics when feeling sick | 1455 | 0.49 (0.32, 0.74) | 0.0009 |
|  | Antibiotics are available to buy at the pharmacy | 1455 | 0.58 (0.36, 0.94) | 0.0258 |
| *Caregiver*  *perceptions of*  *social norms*  *around antibiotic use*  (ref = sometimes) | Caregiver believes that most of their friends think they should give their child antibiotics for a cold | 1451 | 0.68 (0.43, 1.05) | 0.0843 |
|  | Caregiver believes that most of their friends think they should give their child antibiotics for diarrhea | 1451 | 0.85 (0.54, 1.33) | 0.4644 |
|  | Caregiver believes that most of their friends think they should give their child antibiotics for skin rashes | 1452 | 0.69 (0.44, 1.09) | 0.1157 |
|  | Caregiver believes that most of their friends buy their child antibiotics without a prescription | 1451 | 0.76 (0.49, 1.19) | 0.2288 |
| *Caregiver-reported*  *child illness &*  *antibiotic use*  (ref = no) | Child had diarrhea in the last 7 days | 1451 | 0.56 (0.26, 1.16) | 0.1255 |
|  | Child received medical treatment in last 3 months | 1454 | 0.41 (0.23, 0.72) | 0.0022 |
|  | Child took an antibiotic in last 3 months | 1450 | 0.45 (0.23, 0.86) | 0.0164 |
| *Carriage of 3GCR-EC* (ref = no) | 3GCR-EC carriage | 1671 | 0.39 (0.26, 0.6) | <0.00001 |
| *Phenotypic*  *resistance among*  *3GCR-EC isolates*  (ref = susceptible) | Ampicillin (AM) | 772 | 0.15 (0.01, 2.28) | 0.1655 |
|  | Cefotaxime (CTX) | 772 | 0.39 (0.14, 1.13) | 0.0822 |
|  | Cefazolin (CZ) | 772 | - | - |
|  | Ceftazidime (CAZ) | 772 | 0.73 (0.3, 1.67) | 0.4691 |
|  | Cefepime (FEP) | 772 | 2.77 (1.32, 5.73) | 0.0064 |
|  | Imipenem (IPM) | 772 | - | - |
|  | Trimethoprim/sulfamethoxazole (SXT) | 772 | 0.64 (0.32, 1.26) | 0.1925 |
|  | Ciprofloxacin (CIP) | 772 | 0.70 (0.35, 1.40) | 0.3241 |
|  | Gentamicin (GM) | 772 | 0.84 (0.3, 2.16) | 0.7239 |
|  | Tetracycline (TE) | 772 | 0.66 (0.33, 1.33) | 0.2400 |
|  | MDR (3+ classes) | 772 | 0.38 (0.16, 0.91) | 0.0283 |
|  | Total classes | 772 | -0.10 (-0.27, 0.07) | 0.2377 |
| *Abundance of ARGs* | Total ARGs (ref = no change) | 515 | -0.2 (-0.33, -0.07) | 0.0032 |

* Ratios of Adjusted Prevalence Ratios (RaPR) estimated with logistic regressions (Poisson for Total Classes and Total ARGs with absolute difference in difference presented as point estimate) using Generalized Estimating Equations to adjust for repeated measures, and adjustment for confounding by including the following covariates: child age, child sex, household wealth (asset score), caregiver education, and parish. (- indicates model did not converge due to small numbers.) 95% CI: 95% Confidence Intervals. AM: ampicillin; CAZ: ceftazidime; CIP: ciprofloxacin; CTX: cefotaxime; CZ: cefazolin; FEP: cefepime; GM: gentamicin; IPM: imipenem; SXT: trimethoprim/sulfamethoxazole; TE: tetracycline. MDR: multidrug resistant (3+ classes).

####

#### Table S7. Prevalence of *E. coli* sequence types (STs) among sequenced 3GCR-EC isolates before (n=90) and after (n=45) the COVID-19 pandemic began.

| **ST** | **Pre (2019)**  *n (%)* | **Post (2021)**  *n (%)* | *Cont.* | **ST** | **Pre (2019)**  *n (%)* | **Post (2021)**  *n (%)* |
| --- | --- | --- | --- | --- | --- | --- |
| - | 9 (10) | 5 (11.11) |  | 206 | 1 (1.11) | 0 |
| 155 | 5 (5.56) | 2 (4.44) |  | 219 | 0 | 1 (2.22) |
| 394 | 5 (5.56) | 2 (4.44) |  | 226 | 1 (1.11) | 0 |
| 10 | 4 (4.44) | 3 (6.67) |  | 2485 | 0 | 1 (2.22) |
| 131 | 4 (4.44) | 1 (2.22) |  | 29 | 1 (1.11) | 0 |
| 354 | 4 (4.44) | 2 (4.44) |  | 2914 | 1 (1.11) | 0 |
| 5041 | 0 | 4 (8.89) |  | 3346 | 0 | 1 (2.22) |
| 117 | 3 (3.33) | 0 |  | 349 | 1 (1.11) | 1 (2.22) |
| 156 | 3 (3.33) | 0 |  | 428 | 0 | 1 (2.22) |
| 2847 | 3 (3.33) | 1 (2.22) |  | 43 | 1 (1.11) | 0 |
| 38 | 3 (3.33) | 0 |  | 4375 | 0 | 1 (2.22) |
| 93 | 3 (3.33) | 0 |  | 449 | 1 (1.11) | 0 |
| 162 | 2 (2.22) | 1 (2.22) |  | 4541 | 0 | 1 (2.22) |
| 23 | 2 (2.22) | 1 (2.22) |  | 5229 | 1 (1.11) | 0 |
| 2624 | 2 (2.22) | 0 |  | 5236 | 1 (1.11) | 0 |
| 34 | 2 (2.22) | 2 (4.44) |  | 542 | 0 | 1 (2.22) |
| 345 | 2 (2.22) | 0 |  | 58 | 1 (1.11) | 0 |
| 48 | 2 (2.22) | 2 (4.44) |  | 609 | 1 (1.11) | 0 |
| 484 | 0 | 2 (4.44) |  | 616 | 1 (1.11) | 0 |
| 602 | 2 (2.22) | 0 |  | 617 | 1 (1.11) | 0 |
| 101 | 1 (1.11) | 0 |  | 624 | 0 | 1 (2.22) |
| 1101 | 0 | 1 (2.22) |  | 6746 | 1 (1.11) | 1 (2.22) |
| 1137 | 1 (1.11) | 0 |  | 683 | 1 (1.11) | 0 |
| 1140 | 1 (1.11) | 1 (2.22) |  | 69 | 1 (1.11) | 1 (2.22) |
| 1158 | 1 (1.11) | 0 |  | 7285 | 1 (1.11) | 0 |
| 1196 | 0 | 1 (2.22) |  | 744 | 1 (1.11) | 0 |
| 1594 | 1 (1.11) | 0 |  | 752 | 1 (1.11) | 0 |
| 165 | 1 (1.11) | 0 |  | 847 | 1 (1.11) | 0 |
| 1722 | 1 (1.11) | 1 (2.22) |  | 88 | 1 (1.11) | 0 |
| 189 | 1 (1.11) | 0 |  | 90 | 0 | 1 (2.22) |
| 200 | 0 | 1 (2.22) |  | 937 | 1 (1.11) | 0 |
